# Danshen improves survival of patients with advanced lung cancer and targeting the relationship between macrophages and lung cancer cells

**DOI:** 10.18632/oncotarget.18767

**Published:** 2017-06-28

**Authors:** Ching-Yuan Wu, Jong-Yuh Cherng, Yao-Hsu Yang, Chun-Liang Lin, Feng-Che Kuan, Yin-Yin Lin, Yu-Shih Lin, Li-Hsin Shu, Yu-Ching Cheng, Hung Te Liu, Ming-Chu Lu, Jthau Lung, Pau-Chung Chen, Hui Kuan Lin, Kuan-Der Lee, Ying-Huang Tsai

**Affiliations:** ^1^ Department of Chinese Medicine, Chiayi Chang Gung Memorial Hospital, Chiayi, Taiwan; ^2^ School of Chinese medicine, College of Medicine, Chang Gung University, Tao-Yuan, Taiwan; ^3^ Department of Chemistry and Biochemistry, National Chung Cheng University, Taiwan; ^4^ Departments of Nephrology, Chiayi Chang Gung Memorial Hospital, Chiayi, Taiwan; ^5^ Kidney and Diabetic Complications Research Team (KDCRT), Chiayi Chang Gung Memorial Hospital, Chiayi, Taiwan; ^6^ Department of Hematology and oncology, Chiayi Chang Gung Memorial Hospital, Chiayi, Taiwan; ^7^ Department of Pharmacy, Chiayi Chang Gung Memorial Hospital, Chiayi, Taiwan; ^8^ Department of Medical Research and Development, Chang Gung Memorial Hospital, Chiayi branch, Taiwan; ^9^ Institute of Occupational Medicine and Industrial Hygiene, National Taiwan University College of Public Health, Taipei, Taiwan; ^10^ Department of Molecular and Cellular Oncology, The University of Texas MD Anderson Cancer Center, Houston, TX, USA; ^11^ Department of Cancer Biology, Wake Forest University School of Medicine, Medical Center Blvd, Winston-Salem, NC, USA; ^12^ Graduate Institute of Basic Medical Science, China Medical University, Taichung, Taiwan; ^13^ Department of Biotechnology, Asia University, Taichung, Taiwan; ^14^ Division of Pulmonary and Critical Care Medicine of Chang Gung Memorial Hospital, Chiayi, Taiwan, Department of Respiratory Therapy, Chang Gung University, Taoyuan, Taiwan; ^15^ Chang-Gung University College of Medicine, Taoyuan, Taiwan; ^16^ Division of Hematology and Oncology, Department of Internal Medicine, Taipei Medical University Hospital, Taiwan; ^17^ Department of Environmental and Occupational Medicine, National Taiwan University Hospital and National Taiwan University College of Medicine, Taipei, Taiwan

**Keywords:** dihydroisotanshinone I, macrophage, lung cancer, Skp2, CCL2

## Abstract

In traditional Chinese medicine, Salvia miltiorrhiza Bunge (danshen) is widely used in the treatment of numerous cancers. However, its clinical effort and mechanism in the treatment of advanced lung cancer are unclear. In our study, the *in vivo* protective effort of danshen in patients with advanced lung cancer were validated using data from the National Health Insurance Research Database in Taiwan. We observed *in vitro* that dihydroisotanshinone I (DT), a bioactive compound in danshen, exerts anticancer effects through many pathways. First, 10 μM DT substantially inhibited the migration ability of lung cancer cells in both macrophage and macrophage/lung cancer direct mixed coculture media. Second, 10 μM DT repressed the phosphorylation of signal transducer and activator of transcription 3 (STAT3), the protein expression of S-phase kinase associated protein-2 (Skp2), and the mRNA levels of STAT3-related genes, including chemokine (C–C motif) ligand 2 (CCL2). In addition, 10 μM DT suppressed the macrophage recruitment ability of lung cancer cells by reducing CCL2 secretion from both macrophages and lung cancer cells. Third, 20 μM DT induced apoptosis in lung cancer cells. Furthermore, DT treatment significantly inhibited the final tumor volume in a xenograft nude mouse model. In conclusion, danshen exerts protective efforts in patients with advanced lung cancer. These effects can be attributed to DT-mediated interruption of the cross talk between lung cancer cells and macrophages and blocking of lung cancer cell proliferation.

## INTRODUCTION

Lung cancer is the second common malignant disease and the leading cause of death in males in the United States [1]. In Taiwan, lung cancer accounted for 19.7% of all cancer deaths in 2012 [2]. The major treatments include surgery, radiotherapy, chemotherapy, and targeted therapy. Approximately half of newly-diagnosed lung cancer cases are at their advanced stages, which refers to the scenario where cancerous cells have spread either locally or to distant regions in the body. An advanced stage in lung cancer refers to non–small-cell lung cancer that is in stage 3B or stage 4 or extensive-stage small-cell lung cancer. In advanced stages, the effects of treatments are limited, and the treatments are palliative.

Traditional Chinese medicine (TCM), including acupuncture, traumatology manipulative therapies, and decoction, is crucial in health care in Taiwan and other Asian and Western countries. Finished herbal products (FHPs), a modern form of decoctions in which herbal formulae and single herbs are concentrated into granulated compounds, are widely prescribed by TCM physicians because of their convenience and quality. The National Health Insurance (NHI) program in Taiwan reimburses claims for FHPs, including single herbs and herbal formulae, in Taiwan. Because the National Health Insurance Research Database (NHIRD) in Taiwan owned the almost complete information of patients, including the clinical drugs and TCM, it is widely used to investigate the clinical effort of these drugs and TCM on patients in Taiwan [3–9]. The dried root of danshen (*Salvia miltiorrhiza Bunge*) is used for treating numerous cardiovascular and endocrine diseases, including coronary artery disease, angina pectoris, hepatitis, cancers and menstrual disorders, in TCM [10]. However, the clinical efforts of danshen in advanced lung cancer treatment remains unclear.

Tumor-associated macrophages (TAMs) are derived from peripheral blood monocytes recruited into the tumors. The tumor-promoting functions of macrophages at the primary site include supporting tumor-associated angiogenesis and promoting tumor cell invasion, migration, and intra-vasation. Previous studies also showed the association between TAMs and poor prognosis in non–small cell lung cancer [11–13]. In mechanism, TAMs may provide a microenvironment for the invasion and progression of non–small cell lung cancer [14]. Many evidences indicate that macrophages are educated by the tumor microenvironment, so that they can stimulate metastasis of tumor through releasing many compounds, including cytokines [15]. Chemokine (C-C motif) ligand 2 (CCL2), formerly known as monocyte chemoattractant protein-1, was first identified by its ability to attract monocytes *in vitro* [16, 17]. In lung cancer, CCL2 signaling pathway is the important mechanism that TAMs can activate the growth and metastasis of lung cancer cells through the bidirectional cross talk between macrophages and lung cancer cells [18]. Therefore, blocking the CCL2 signaling pathway may prove beneficial for halting lung cancer progression.

In this study, we aimed to examine the protective efforts of danshen in advanced lung cancer. First, we analyzed the advanced lung cancer by using the National Health Insurance Research Database (NHIRD) in Taiwan to validate the protective efforts of danshen *in vivo*. *In vitro*, we found that dihydroisotanshinone I (DT, Supplementary Figure 2A), the bioactive compound present in danshen, can inhibit the migration of lung cancer cells in lower concentration (10 μM). Furthermore, we found that 10 μM DT can suppress the phosphorylation of STAT3 (signal transducer and activator of transcription 3) and the protein expression of Skp2 (S-phase kinase associated protein-2) and the mRNA levels of their down-stream genes. We also clarified the inhibitory effect of 10 μM DT on the interaction between macrophages and lung cancer cells through blocking the CCL2 pathway. Moreover, we discovered that the higher concentration (20 μM) of DT can induce the apoptosis of the lung cancer cells. Moreover, we found that DT treatment (30 mg/kg) significantly inhibited the final tumor volume on xenograft nude mice. These results suggest that DT might be a novel anticancer agent for advanced lung cancer treatment.

## RESULTS

### Protective effort of danshen in advanced stages of lung cancer patients from Taiwan

We included a total of 60,267 patients (20,645 women and 39,622 men) diagnosed with late stage of lung cancer during the study period (Supplementary Figure 1). The basic demographic characteristics of the patient population are summarized in Table 1. In study cohort, the patients tended to be elderly, male and have a high CCI. The crude Cox regression analysis demonstrated a strong association between the use of danshen and a decrease in mortality (Table 1). Compared with danshen nonusers or used < 30 g of danshen, danshen users who had used ≥ 90 g had reduced mortality by 58.4% (crude HR, 0.416; 95% CI, 0.255–0.680 [*p* < 0.0001]). The group who had used < 90 g and ≥ 30 g of danshen had reduced mortality by 63.7% (crude HR, 0.363; 95% CI, 0.296–0.812 [*p* < 0.0001]). On the multivariate Cox model controlling for age, gender, income, urbanization, Charlson comorbidity index and other drug use (cisplatin, carboplatin, erlotinib and gefitinib), the use of danshen remained highly associated with decreased mortality (the adjusted HR of danshen users who had used ≥ 90 g was 0.571 [95% CI, 0.349–0.932] (*p* = 0.025) and the adjusted HR of danshen users who had used < 90 g and ≥ 30 g was 0.480 [95% CI, 0.306–0.753] (*p* = 0.001) (Table 1). For the 1:4 matched cohort, the crude cox regression analysis also demonstrated a strong association between the use of danshen and a decrease in mortality (Table 2). Compared with danshen nonusers or used < 30 g of danshen, danshen users who had used ≥ 90 g had reduced mortality by 50.9% (crude HR, 0.491; 95% CI, 0.296–0.812 [*p* = 0.006]). The group who had used < 90 g and ≥ 30 g of danshen had reduced mortality by 57.1% (crude HR, 0.429; 95% CI, 0.270–0.683 [*p* < 0.0001]). On the multivariate Cox model analysis, the use of danshen remained highly associated with decreased mortality (the adjusted HR of danshen users who had used ≥ 90 g was 0.541 [95% CI, 0.326–0.897] (*p* = 0.017) and the adjusted HR of danshen users who had used < 90 g and ≥ 30 g was 0.470 [95% CI, 0.295–0.749] (*p* = 0.002) (Table 2). The trend of relationship between danshen use and the risk reduction of mortality did not alter when the matched cohort was used. Notably, the reduced mortality between those who had used ≥ 90 g of danshen and those who had used < 90 g and ≥ 30 g of danshen don’t show significant difference in both the study cohort and the 1:4 matched cohort. It is possible that the smaller size of the patients those who had used ≥ 90 g of danshen (*N* = 300) and the group who had used < 90 g and ≥ 30 g of danshen (*N* = 408).

**Table 1:** Crude and adjusted hazard ratios (HRs) of mortality during the follow-up period in study cohort

Variables	Frequency		Crude hazard ratios			Adjusted hazard ratios *		
	count	%	Risk ratio	95% CI	*P* value	Risk ratio	95% CI	*P* value
**Gender**								
Female (ref.)	20645	34.26	1.000			1.000		
Male	39622	65.74	1.364	1.295–1.436	0.000*	1.340	1.272–1.412	0.000*
**Age**								
< = 65 (ref.)	19702	32.69	1.000			1.000		
> 65	40565	67.31	1.677	1.587–1.773	0.000*	1.354	1.278–1.434	0.000*
Charlson Comorbidity Index								
< = 6 (ref.)	23339	38.73	1.000			1.000		
> 6	36928	61.27	1.383	1.315–1.453	0.000*	1.567	1.490–1.648	0.000*
**Danshen1**								
0 (ref.)	59559	98.83	1.000			1.000		
1	408	0.68	0.363	0.232–0.570	0.000*	0.480	0.306–0.753	0.001*
2	300	0.50	0.416	0.255–0.680	0.000*	0.571	0.349–0.932	0.025*
**Covariates**								
**Cisplatin**								
No (ref.)	41370	68.64	1.000			1.000		
Yes	18897	31.36	0.421	0.395–0.448	0.000*	0.494	0.463–0.528	0.000*
**Carboplatin**								
No (ref.)	57698	95.74	1.000			1.000		
Yes	2569	4.26	0.563	0.486–0.654	0.000*	0.680	0.586–0.790	0.000*
**Erlotinib**								
No (ref.)	58871	97.68	1.000			1.000		
Yes	1396	2.32	0.311	0.237–0.407	0.000*	0.504	0.384–0.662	0.000*
**Gefitinib**								
No (ref.)	57163	94.85	1.000			1.000		
Yes	3104	5.15	0.287	0.238–0.345	0.000*	0.489	0.405–0.591	0.000*

*adjusted for age, gender, income, urbanization, charlson comorbidity index and other drug use (cisplatin, carboplatin, erlotinib, gefitinib).

1. Danshen total quantity > = 90 g is “2”, 30 g < = Danshen total quantity < 90 g is “1”, 30 g > Danshen total quantity = 0 g is “0”.

**Table 2:** Crude and adjusted hazard ratios (HRs) of mortality during the follow-up period in 1:4Matched cohort

Variables	Frequency		Crude hazard ratios			Adjusted hazard ratios *		
	count	%	Risk ratio	95% CI	*P* value	Risk ratio	95% CI	*P* value
**Gender**								
Female (ref.)	1615	45.62	1.000			1.000		
Male	1925	54.38	1.426	1.137–1.789	0.002*	1.316	1.043–1.662	0.021*
**Age**								
< = 6 5 (ref.)	1625	45.90	1.000			1.000		
> 65	1915	54.10	1.781	1.410–2.250	0.000*	1.466	1.143–1.879	0.003*
Charlson Comorbidity Index								
<=6 (ref.)	2000	56.50	1.000			1.000		
> 6	1540	43.50	1.166	0.936–1.452	0.171	1.360	1.088–1.699	0.007*
**Danshen1**								
0 (ref.)	2832	80.00	1.000			1.000		
1	408	11.53	0.429	0.270–0.683	0.000*	0.470	0.295–0.749	0.002*
2	300	8.47	0.491	0.296–0.812	0.006*	0.541	0.326–0.897	0.017*
**Covariates**								
**Cisplatin**								
No (ref.)	2275	64.27	1.000			1.000		
Yes	1265	35.73	0.473	0.361–0.619	0.000*	0.587	0.438–0.786	0.000*
**Carboplatin**								
No (ref.)	3379	95.45	1.000			1.000		
Yes	161	4.55	0.198	0.063–0.617	0.005*	0.241	0.077–0.752	0.014*
**Erlotinib**								
No (ref.)	3438	97.12	1.000			1.000		
Yes	102	2.88	0.569	0.235–1.377	0.211	0.824	0.337–2.017	0.671
**Gefitinib**								
No (ref.)	3284	92.77	1.000			1.000		
Yes	256	7.23	0.376	0.194–0.729	0.004*	0.688	0.346–1.372	0.288

*adjusted for age, gender, income, urbanization, charlson comorbidity index and other drug use (cisplatin, carboplatin, erlotinib, gefitinib).

1. Danshen total quantity > = 90 g is “2”, 30 g < = Danshen total quantity < 90 g is “1”, 30 g > Danshen total quantity = 0 g is “0”.

### Inhibition of DT on cell motility in various human lung cancer cells

**Figure 1:**
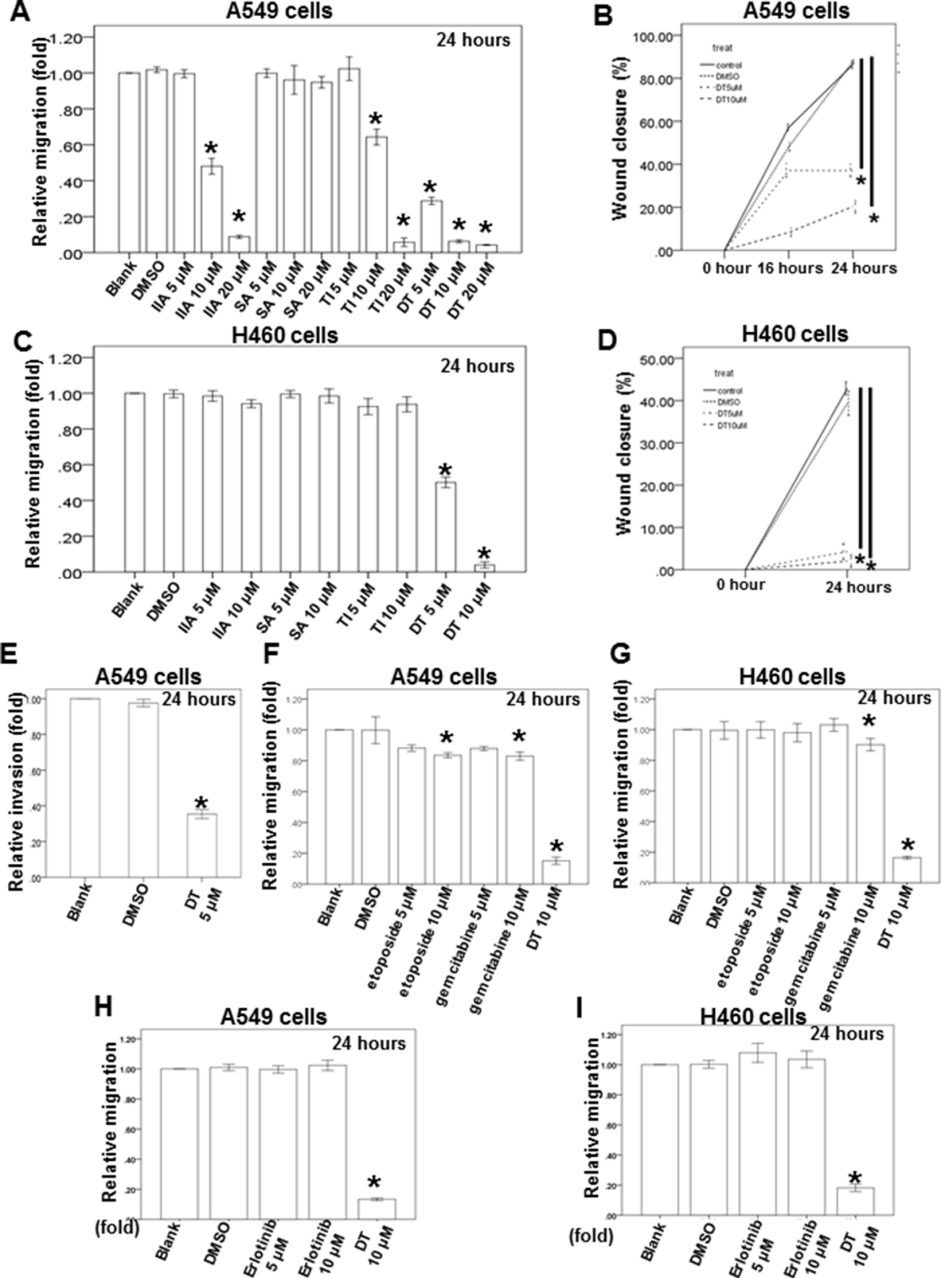
Dihydroisotanshinone I block different human lung cancer cells migration on *in vitro* transwell migration assay, wound healing assay and invasion assay The migration ability of A549 cells or H460 cells were measured by the transwell migration assay. After treated with indicated drugs for 24 hours, the photographs (× 100) were taken and the migratory cells were measured using AlphaEase^®^FC StandAlone Software. Numbers of the migratory A549 cells (**A**, **F**, **H**) and H460 cells (**C**, **G**, **I**) in each group were normalized to the control. The mobility of lung cancer cells were measured by wound-healing assay. After treatment with indicated drugs, photographs (×100) were taken. The wound closure of A549 cells (**B**) and H460 cells (**D**) were quantified by measuring the remaining unmigrated area using AlphaEase^®^FC StandAlone Software. The invasion ability of A549 cells, were measured by the transwell invasion assay. After treated without or with DMSO or DT for 24 hours, the photographs (× 100) were taken and the invasive cells were measured using AlphaEase^®^FC StandAlone Software. Numbers of the invasive A549 cells (**E**) in each group were normalized to the control. The results were from three independent experiments. (Error bar = mean ± S.E.M. Asterisks (*) mark samples significantly different from blank group with *p* < 0.05).

In previous data, we showed the protect effort of danshen for the advanced lung cancer patients in Taiwan. Depending on their structure and properties, there are at least more than 50 compounds isolated from danshen [19]. In general, these compounds have been divided into two groups. The first group is phenolic acids, such as salvianolic acid B (SA), and their structure contain caffeic acid monomers and oligomers. The second group is tanshinones, such as tanshinone I (TI), tanshinone IIA (IIA) and dihydroisotanshinone I (DT), and their structure contain abietane diterpenes with a common ortho- or para-naphthoquinone chromophore (Supplementary Figure 2A). To study the effect of these compounds on migration ability of lung cancer cells, we used the human lung adenocarcinoma cell line (A549 cells) and human lung large cell carcinoma cell line (H460 cells) as our model to investigate the effect of these compounds, including SA, TI, IIA and DT, in the migration assay and the healing assay (Figure 1A–1D). After treatment with indicated compounds for an indicated number of hours, our results showed that 10–20 μM tanshinones, including TI, IIA and DT, significantly inhibited the migratory ability of A549 cells in a dose-dependent manner (Figure 1A, 1B). Moreover, DT had the best inhibitory effect on the migratory ability of A549 cells even in the lower concentration (5 μM). Next, we also discovered that the 5–10 μM DT had the similar inhibitory effect on the migratory ability of H460 cells (Figure 1C, 1D). We also discovered that lower concentration (5 μM) DT can block the invasion of A549 cells (Figure 1E). To compare the effect of DT with the clinical common used agents for advanced lung cancer, we investigated the effect of the anti-lung cancer agents, including gemcitabine hydrochloride, etoposide and erlotinib, on the migration ability of A549 cells and H460 cells. Our results showed that 10 μM gemcitabine hydrochloride or etoposide only mildly inhibited the migration ability of lung cancer cells in indicated hours, except 10 μM DT (Figure 1F–1I). These data suggest DT can inhibit the migration of lung cancer cells.

### Effects of DT on lung cancer cells migration with the macrophage medium or the lung cancer/macrophages co-culture *in vitro* model and on RAW 264.7 cell recruitment *in vitro*

**Figure 2:**
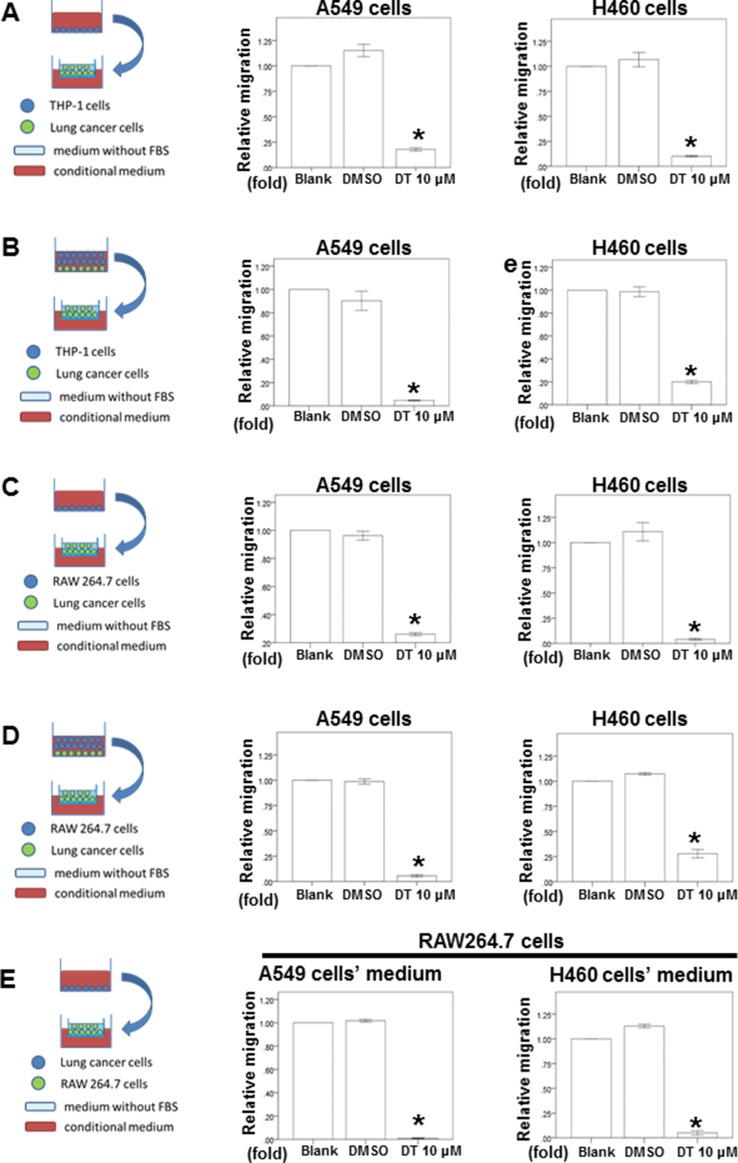
The effect of DT on lung cancer cells migration in macrophages medium or the lung cancer/macrophages co-culture *in vitro* model and on RAW 264.7 cells recruitment *in vitro* model The migration ability of human lung cancers in the macrophages medium or the lung cancer/macrophages co-culture *in vitro* model were measured by the transwell migration assay. THP1 cells (**A**) or RAW 264.7 cells (**C**) were treated with indicated drugs for 24 hours. Then the conditioned medium was collected and placed in the lower chamber. The lung cancer cells were then placed on the upper chamber for the migration assay. After incubation for 16 hours, the photographs (× 100) were taken and the migratory cells were measured using AlphaEase^®^FC StandAlone Software. The quantification of the indicated migratory lung cancer cells numbers in each group were normalized to the control. In the co-culture *in vitro* model, THP1 cells (**B**) or RAW 264.7 cells (**D**) and the indicated human lung cancers were directly mix co-cultured and treated with indicated drugs for 16 hours. Then the conditioned medium were collected and placed in the lower chamber. The indicated lung cancer cells were then placed on the upper chamber for the migration assay. After incubation for 16 hours, the photographs (× 100) were taken and the migratory cells were measured using AlphaEase^®^FC StandAlone Software. The quantification of the migratory indicated lung cancer cells numbers in each group were normalized to the control. For the macrophages’ recruitment ability of human lung cancer cells, the A549 cells were treated with indicated drugs for 24 hours. Then the conditioned medium were collected and placed in the lower chamber. The RAW 264.7 cells were then placed on the upper chamber for the migration assay. After incubation for 24 hours, the photographs (× 100) were taken and the migratory cells were measured using AlphaEase^®^FC StandAlone Software. The quantification of the migratory RAW 264.7 cells numbers in each group were normalized to the control (**E**). The results were from three independent experiments. (Error bar = mean ± S.E.M. Asterisks (*) mark samples significantly different from blank group with *p* < 0.05).

Previous study has demonstrated that macrophages can promote lung tumor invasion and metastasis [12, 20]. Because 10 μM of DT have the inhibitory effect in the cells motility of both A549 cells and H460 cells, we next investigated the effect of DT on the ability of macrophages to promote tumor migration in 10 μM (Figure 2). After THP-1 cells (Figure 2A) or RAW 264.7 cells (Figure 2C) were treated without or with DMSO or 10 μM DT for 24 hours, the conditioned medium was collected and placed in the lower chambers of transwell plates. The lung cancer cells were then placed in the upper chambers of transwell plates with inserts in a serum-free medium for a migration assay. Our results showed that 10 μM DT significantly inhibited the migration of A549 cells and H460 cells in the THP-1 cells (Figure 2A) or RAW 264.7 cells medium (Figure 2C). In previous studies, direct mixed coculture of macrophages and tumor cells led to stronger activation of signaling by transcription factors than did indirect separate coculture in transwell chamber dishes [21, 22]. In the direct mixed coculture system, cells communicate with each other through direct contact, which more closely approximates the physiological situation. Therefore, we examined the migratory ability of lung cancer under DT treatment in the direct mixed coculture of lung cancer cells and THP-1 cells (Figure 2B) or RAW 264.7 cells *in vitro* (Figure 2D). Our results showed that 10 μM DT significantly inhibited the migration ability of lung cancer cells in the direct mixed coculture medium (Figure 2B, 2D). More evidences indicates that macrophages can migrate into tumors and accumulate in distinct tumor microenvironments depending on the chemokine expression pattern [20, 23–25]. For this, we investigated the effect of macrophage recruitment by DT-treated lung cancer cells. Lung cancer cells were treated with the indicated drugs for 24 hours. The conditioned media were collected and then placed in the lower chambers of transwell plates. The RAW 264.7 cells were then placed in the upper chambers of transwell plates in serum-free medium for a migration assay (Figure 2E). These tests indicated that A549 cells or H460 cells can induce RAW 264.7 cell migration with or without DMSO treatment. In addition, we found that 10 μM DT inhibited the migration of RAW 264.7 cells induced by A549 cells or H460 cells (Figure 2E). These data suggest that DT can block the ability of macrophages to promote lung cancer migration and block the macrophage recruitment ability of lung cancer cells.

### DT-induced inhibition of cytokine secretion from lung cancer cells and macrophages

**Figure 3:**
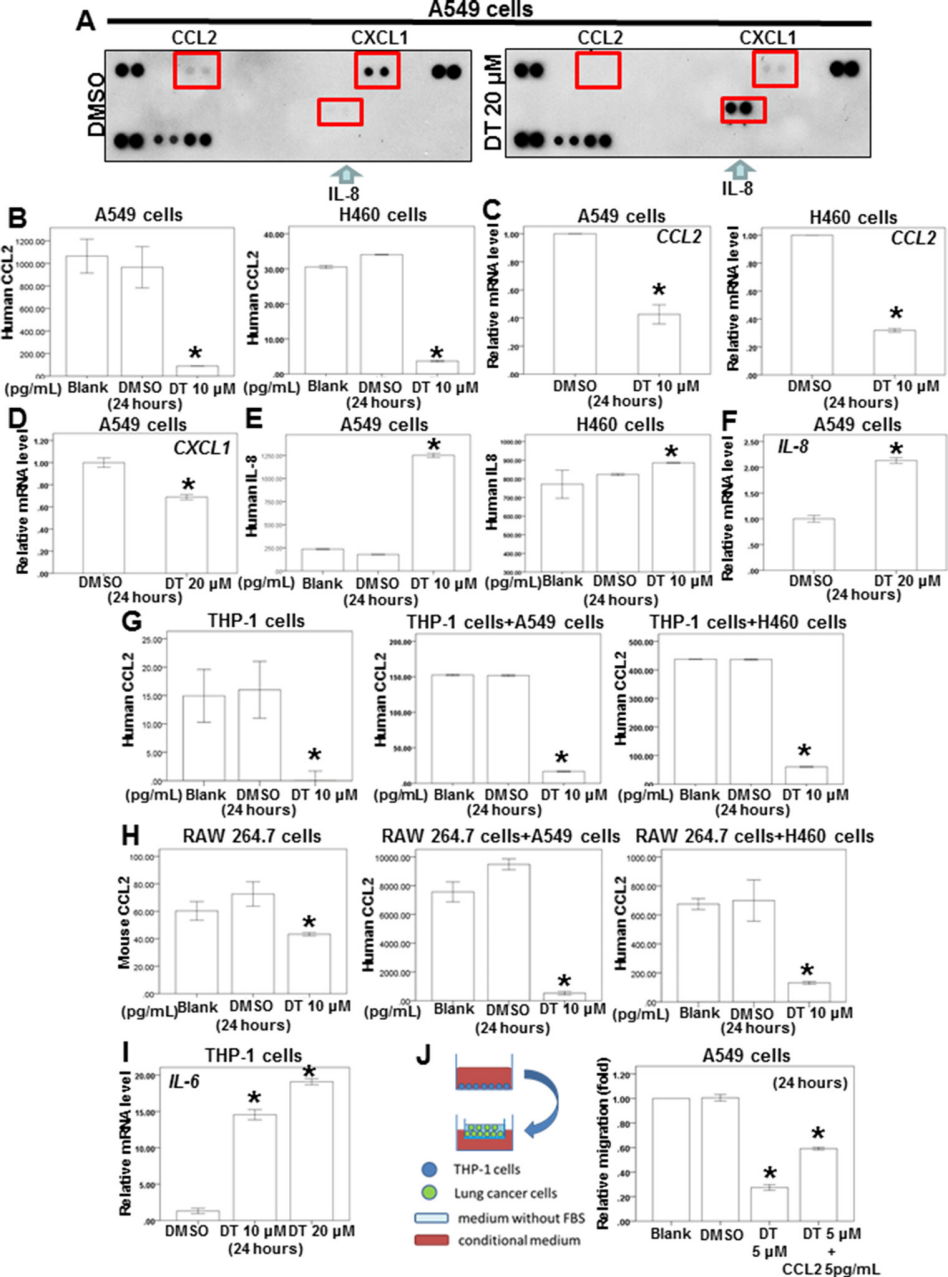
The effect of DT on the proteins secretion from lung cancer cells and macrophages co-culture *in vitro* (**A**) For cytokines array, A549 cells were treated with DMSO or DT 20 μM for 24 hours. The cultured medium was collected and analyzed by cytokines microarray. Array images were captured following 5-min exposure to X-ray film. (**B**, **E**, **G**, **H**) For ELISA, the conditioned medium of A549 cells or H460 cells (B, E) or THP1 cells or RAW 264.7 cells or coculture with THP1 cells/lung cancer cells or RAW 264.7 cells/lung cancer cells (G, H) were collected from untreated cells and cells treated with DMSO or indicated drugs for 24 hours. The secretion of human or mouse CCL2 or human IL-8 were measured by ELISA kits. (**C**, **D**, **F**, **I**) Total mRNA was extracted from the A549 cells or H460 cells (C, D, F) or THP-1 cells (I) after treat without or with indicated drugs for 24 hours. The coding regions of human *IL-6, IL-8, CXCL-1* and *CCL2* were used as probes for real time polymerase chain reaction analysis. (**J**) The migration ability of human lung cancers in the macrophages medium *in vitro* model were measured by the transwell migration assay. THP1 cells were treated with DMSO or DT for 24 hours. Then the conditioned medium was collected and placed in the lower chamber. In the group of DT+ 5 pg/mL CCL2, 5 pg/mL CCL2 was added into the condition medium of this group. Then, A549 cells were then placed on the upper chamber for the migration assay. After incubation for 24 hours, the photographs (× 100) were taken and the migratory cells were measured using AlphaEase^®^FC StandAlone Software. The quantification of the indicated migratory lung cancer cells numbers in each group were normalized to the control. All the results are representative of at least three independent experiments. (Error bars = mean ± S.E.M. Asterisks (*) mark samples significantly different from blank group with *p* < 0.05).

Substantial evidence has indicated that macrophages are modulated in the tumor microenvironment to promote metastatic processes by producing many compounds, including cytokines [15]. Notably, tumor cells secrete some molecules, such as chemokines, during invasion and migration. Our results indicated that DT inhibits lung cancer cell mobility. In addition, DT inhibits the macrophage recruitment ability of lung cancer cells. Because 20 μM DT had the highest inhibitory effect on the migration ability of A549 cells (Figure 1A), we screened the secreted cytokines from DMSO- or 20-μM-DT-treated A549 cells by using a human cytokines array. After 24 hours, DT treatment resulted in decreased expression of CCL2 and C–X–C motif chemokine ligand 1 (CXCL1) and increased expression of IL-8 in the cytokine profile (Figure 3A). A previous study demonstrated increased CCL2 levels in lung cancer patients with bone metastases compared with patients with localized tumors [26]. We examined the effects of DT on the secretion of the aforementioned cytokines from lung cancer cells or macrophages through ELISA and qPCR to confirm the results of the cytokine array. After treatment of A549 and H460 cells with 10 μM DT for 24 hours, we observed that CCL2 secretion and *CCL2* mRNA expression were significantly inhibited (Figure 3B, 3C). In addition, *CXCL1* mRNA expression was inhibited after treatment of A549 cells with 20 μM DT (Figure 3D). The ELISA results revealed that IL-8 secretion was increased in A549 and H460 cells after 10 μM DT treatment (Figure 3E). Furthermore, the qPCR results indicated that the mRNA expression of IL-8 increased after DT treatment of A549 cells (Figure 3F). *CCL2* and *CXCL1* are the downstream target genes of activated STAT3 [27–30]. In addition, activated STAT3 can occupy the endogenous IL-8 promoter and directly inhibit IL-8 transcription and expression [31, 32]. These results suggested that the STAT3 signaling pathway may be the mechanism through which DT treatment regulates cytokines. For macrophages, we observed that DT also inhibited CCL2 secretion from both THP-1 and RAW 264.7 cells (Figure 3G, 3H). An *in vitro* ELISA analysis demonstrated that DT treatment inhibited CCL2 secretion from a lung cancer cell and macrophage coculture (Figure 3G, 3H). CCL2 is a cytokine biomarker of M2 macrophages [33, 34]. DT treatment reduced the CCL2 secretion of THP-1 cells (Figure 3G). The results suggested that THP-1 cells can polarize to M1 macrophages under DT treatment. Because IL-6 is a biomarker of M1 macrophages [35], increased *IL-6* mRNA expression was observed in THP-1 cells (Figure 3I), suggesting that DT treatment can induce THP-1 cells to differentiate into M1 macrophages. To validate the critical role of CCL2 in controlling the migration ability of DT-treated lung cancer cells, we investigated the effects of DT with or without CCL2 on the migration ability of macrophages. Our results revealed that 5 μM DT significantly inhibited the migration ability of A549 cells in the THP-1 cell medium (Figure 3J). After adding 5 pg/mL of CCL2 to the conditioned medium, we observed that CCL2 partially rescued the migration ability of DT-treated A549 cells (relative migration: from 25% to 60%). The human cytokine array showed that DT treatment can inhibit both CCL2 and CXCL1 expression (Figure 3A). Previous studies have reported CXCL1 as an important cytokine in lung cancer development [36, 37]. Our results suggested that CCL2 is one of the critical cytokines that control the migration ability of DT-treated lung cancer cells. Altogether, our data suggested that DT inhibits the migration of both macrophages and lung cancer cells by blocking the expression of several cytokines, and CCL2 is one of these critical cytokines under DT treatment.

### Expression of the whole genomic mRNA and lncRNA profiling of A549 cells treated with DT

**Figure 4:**
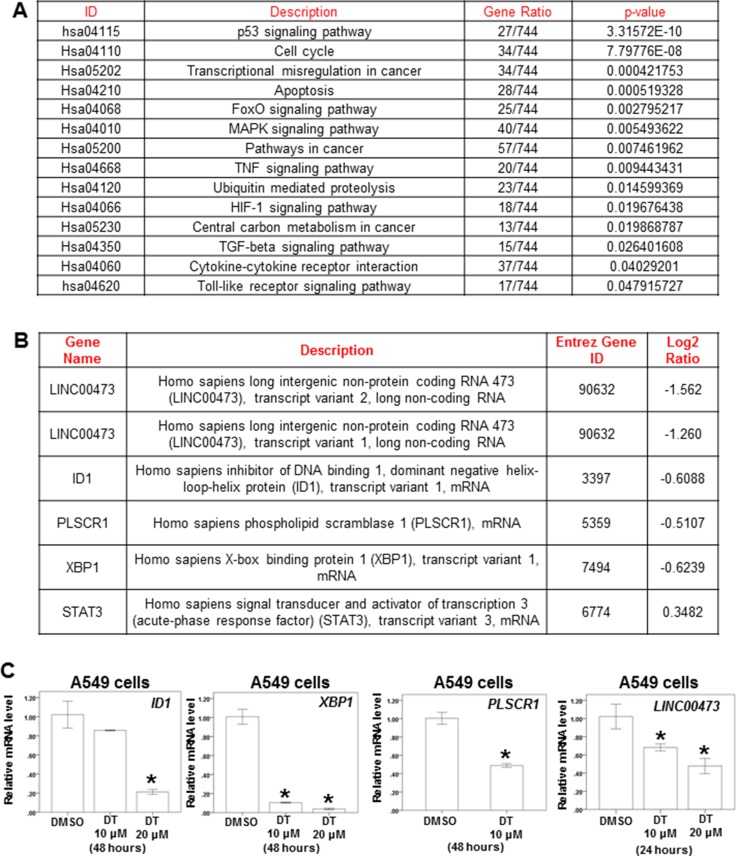
The effect of DT on the whole genomic mRNA and lncRNA Profiling of A549 cells (**A**) After KEGG enrichment analysis, the pathway in cancer for regulating cancer metastasis and migration was identified. (**B**) The RNA expression of *LINC473, ID1, PLSCR1, XBP1* and *STAT3* were showed through RNA array. (**C**) Total mRNA was extracted from the A549 cells after treat without or with indicated drugs for 24 hours. The coding regions of human *ID1, XBP1, PLSCR1* and *LINC473* were used as probes for real time polymerase chain reaction analysis. (Log2 ratio: the differential expressed level between the A549 cells treated with DMSO or DT. Error bars = mean ± S.E.M. Asterisks (*) mark samples significantly different from blank group with *p* < 0.05).

To further evaluate the pathway maps and molecular and cellular functions of the genes regulated by DT on lung cancer, we analyzed the whole genome mRNA and lncRNA (Long non-coding RNAs) expression profile of A549 cells treated with DMSO or 10 μM DT for 24 hours through mRNA array and lncRNA array. Compared treated with DMSO or 10 μM DT, 1539 up-regulated genes and 810 down-regulated genes were discovered (Supplementary Table 1). Following Kyoto encyclopedia of genes and genomes (KEGG) enrichment analysis, the 30 pathways were identified (Supplementary Table 2, *p* < 0.05). For regulating cancer pathway, there are 14 pathways, including the pathway in cancer (hsa05200; *p*-value < 0.05), apoptosis pathway (hsa04210; *p*-value < 0.001) and transcriptional mis-regulation in cancer (hsa05202; *p*-value < 0.001), was identified through KEGG analysis (Figure 4A and Supplementary Figure 2B, 2C). In the lncRNA array, the 124 lncRNA were involved in A549 cells under the treatment of DT (Supplementary Table 3). In previous study, elevated *LINC00473* (*LINC473*, long intergenic non-protein coding RNA 473, Entrez Gene ID: 90632) expression correlated with poor prognosis in lung cancer and sustained *LINC473* expression was required for the growth and survival of lung cancer cells [38]. The expression of *LINC473* could be increased through phosphorylate STAT3 at Tyr705 [39]. In our data, the expression of *LINC473* was significantly decreased under the treatment of DT in two different transcript variants (NR_026860 and NR_026861) (Figure 4B). We confirmed that the expression of *LINC473* were inhibited by DT in a dose dependent manner through qPCR (Figure 4C). Moreover, we also observed the mRNA expression of several STAT3’s target genes which are reported by Professor Christine Watson [40], including *ID1* (inhibitor of DNA binding 1, dominant negative helix-loop-helix protein, transcript variant 1, log2(Ratio) = −0.6088), *PLSCR1* (phospholipid scramblase 1, log2 (Ratio) = −0.5107) and *XBP1* (X-box binding protein 1, log2 (Ratio) = −0.6239), were also decreased (Figure 4B). We also confirmed that the mRNA expression of *ID1* and *PLSCR1* and *XBP1* were inhibited by DT through qPCR (Figure 4C). These data suggest that DT may involve several pathways in inhibiting the growth and metastasis of A549 cells and STAT3 maybe the one of the critical targets.

### Effects of DT on STAT3 phosphorylation, Skp2 protein expression, and lung cancer cell proliferation

**Figure 5:**
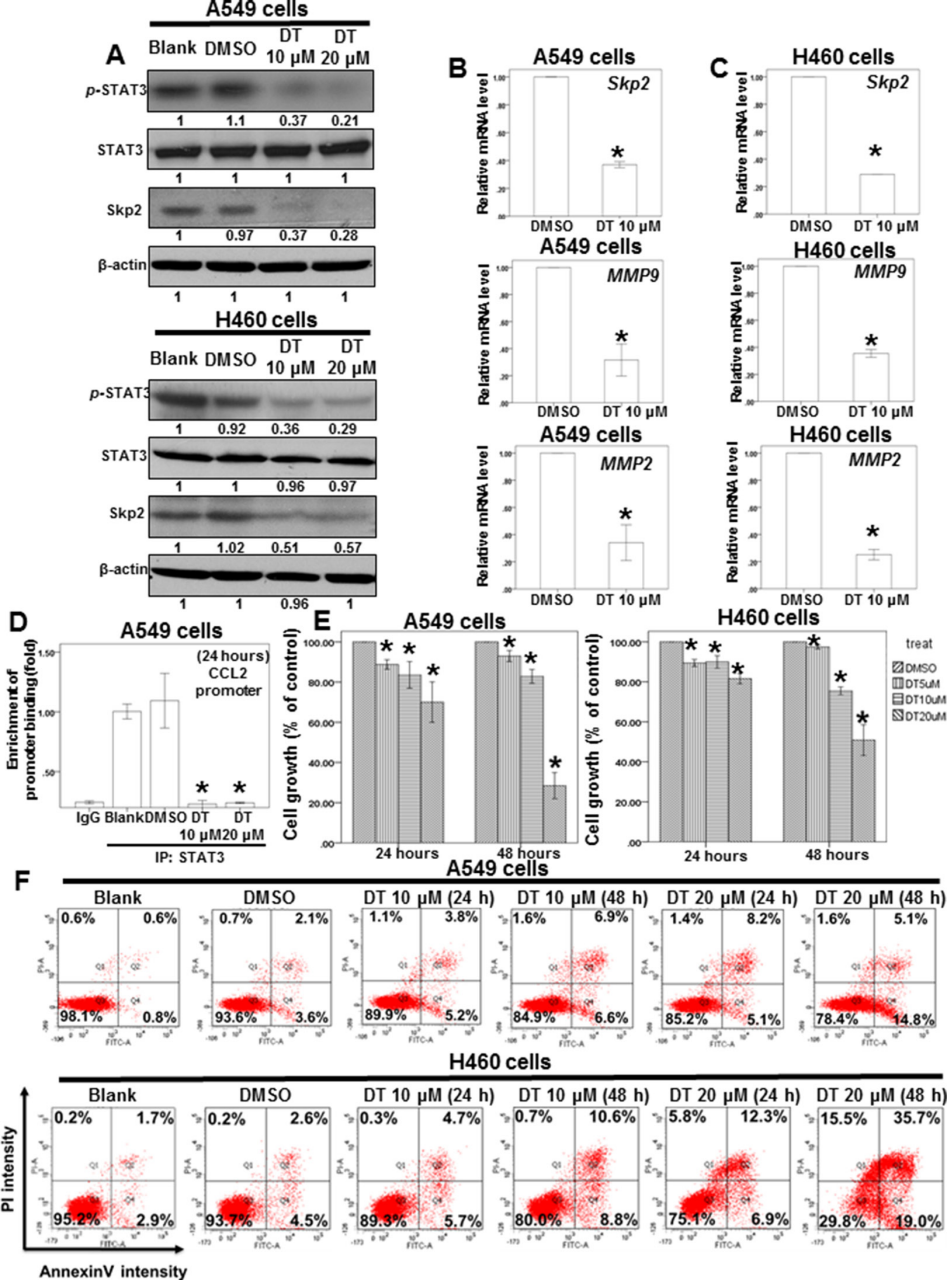
DT inhibit the protein expression of p-STAT3 and Skp2 and mRNA level of its downstream genes and block the proliferation of lung cancer cell lines (**A**) Total cell extracts of A549 cells or H460 cells were harvested from cells treated with DMSO or indicated concentrations of DT for 24 hours. The protein was immunoblotted with polyclonal antibodies specific for *p*-STAT3 or STAT3 or Skp2. β-actin was used as an internal loading control. (**B**, **C**) Total mRNA was extracted from the A549 cells (B) or H460 cells (C) after treat without or with indicated drugs for 24 hours. The coding regions of human *Skp2 MMP2,* and *MMP9* were used as probes for real time polymerase chain reaction analysis. (**D**) ChIP assay for CCL2 promoter in A549 cells treated with indicated concentration of DT. (**E**) A549 cells or H460 cells were measured by XTT assay after 24–48 hours of culturing in the presence of different concentration DT. (**F**) For apoptosis assay, A549 cells or H460 cells were treated with 10–20 μM of DT for 24–48 hours. Cell apoptosis was detected by flow cytometry with annexin-V-FITC/PI dual staining. The representative histograms of flow cytometric analysis using double staining with annexin-V-FITC (FITC-A) and PI (PI-A). Q1 (annexin−V−/PI+) show necrosis cells; Q2 (annexin−V+/PI+) show the late apoptosis cells; Q3 (annexin−V−/PI−) show normal cells; Q4 (annexin−V+/PI−) show the early apoptosis cells. (Error bars = mean ± S.E.M. Asterisks (*) mark samples significantly different from DMSO group with *p* < 0.05).

STAT3 is a critical protein in tumor metastasis, including that of lung cancer [41, 42]. Moreover, STAT3 is a valuable biomarker for prognosis prediction and a therapeutic target in human solid tumors [43]. Our data demonstrated that 10 μM DT significantly reduced the mRNA expression of *STAT3* downstream genes, including *LINC473, ID1, PLSCR1*, and *XBP1*. However, mRNA array results revealed that DT did not inhibit the mRNA levels of *STAT3* (Figure 4B). We investigated the effects of DT on STAT3 Tyr705 phosphorylation (p-STAT3) through a Western blotting assay, which showed that DT (10–20 μM) can inhibit STAT3 phosphorylation in both A549 and H460 cells. Furthermore, in accordance with the mRNA expression of *STAT3*, the protein expression of STAT3 did not change under DT treatment (Figure 5A). Since STAT3 are known to bind the promoter area of *CCL2* gene [44], we performed chromatin immunoprecipitation (ChIP) to examine the binding of STAT3 to CCL2 gene promoters under the treatment of DT. Our ChIP assays revealed that while STAT3 bound in the CCL2 gene promoter in untreated A549 cells, 10–20 μM DT treatment caused a dissociation of STAT3 from the promoter of CCL2 gene (Figure 5D).

**Figure 6:**
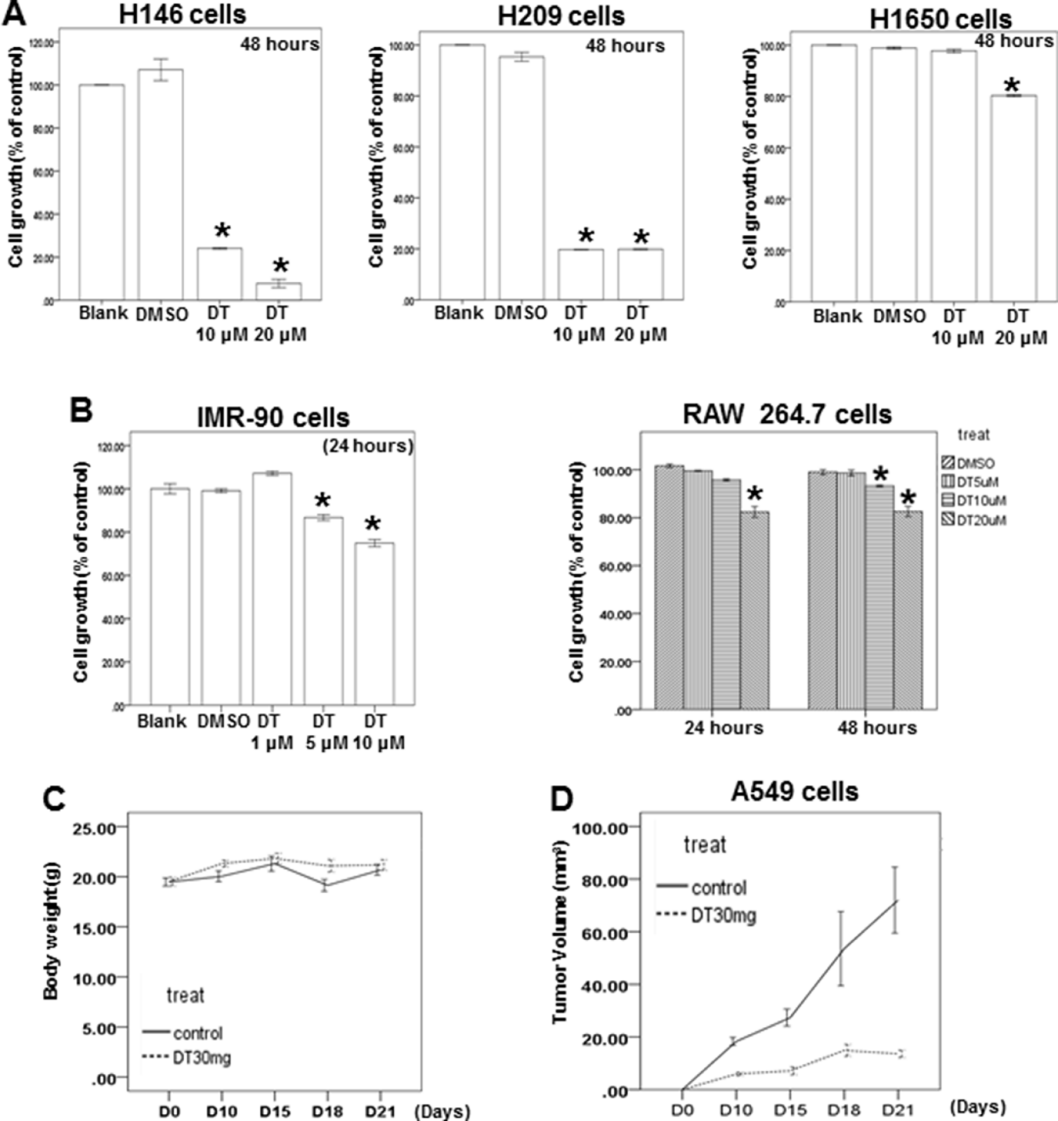
The effect of DT on the different lung cancer cell lines, normal lung fibroblasts, macrophages and xenografted animal model (**A**, **B**) H146 cells, H209 cells, H1650 cells, IMR-90 cells and RAW 264.7 cells were measured by XTT assay after culturing in the presence of indicated concentration DT for indicated hours. (**C**) Average mice weights with every 2-day injection of vehicle/DT over a time course of 3 weeks. (**D**) Average tumor volume of mice injected with either vehicle (DMSO) or DT (30 mg/kg, *n* = 5 per group). (Error bars = mean ± S.E.M. Asterisks (*) mark samples significantly different from DMSO group with *p* < 0.05).

Skp2, a transcriptional target of STAT3 [45], is a vital protein in lung cancer metastasis and proliferation [46–48]. We examined the protein expression of Skp2 in DT-treated lung cancer cells. Western blotting (Figure 5A) and immunofluorescence assays (Supplementary Figure 4) revealed that the protein expression of Skp2 decreased in both A549 and H460 cells under DT treatment. The upregulation of MMP-2 and MMP-9 is one of the mechanisms through which Skp2 promotes lung cancer cell invasion [49]. Furthermore, qPCR showed that the mRNA expression of *Skp2* and downstream genes, including *MMP2* and *MMP9*, was inhibited by treatment of A549 and H460 cells with 10 μM DT (Figure 5B, 5C). Because Skp2 is associated with the aggressiveness and proliferation of lung cancer [46–48], we also determined the effects of DT on lung cancer cell growth. Our results showed that the IC50 values (48 hours) in A549 and H460 cells were 15.487 and 19.389 μM, respectively (Figure 5E). Because the mRNA array demonstrated the involvement of the apoptosis pathway under 10 μM DT treatment (Figure 4A), we investigated whether the DT-induced inhibition of cell proliferation is associated with apoptosis through flow cytometry by using annexin V/PI dual staining. The results showed that that 20 μM DT treatment can induce significant apoptosis in both A549 and H460 cells (approximately 19.9% and 54.7%, respectively, in 48 hours) (Figure 5F). These results suggested that apoptosis may be a major mode of lung cancer cell death induced by an increased DT concentration (approximately 20 μM) in 48 hours. Furthermore, DT inhibited the proliferation of H146, H209 (small lung cancer cell line), and H1650 cells (lung adenocarcinoma cell line with EGFR mutation; Figure 6A). In addition, we investigated the cytotoxicity of DT in normal cells. The proliferation of IMR-90 cells (human normal lung fibroblasts) was mildly inhibited by 5–10 μM DT treatment in 24 hours (Figure 6B). Furthermore, 5–20 μM DT mildly inhibited the proliferation of RAW 264.7 cells in 24–48 hours (Figure 6B). For an *in vivo* study, we investigated the effects of DT on a xenograft nude mouse model. DT treatment (30 mg/kg, IP) significantly inhibited the final tumor volume by approximately 61% in 3 weeks (Figure 6D), whereas DT did not significantly alter either the activity or body weight of mice, and no mice were dead after DT treatment (30 mg/kg, IP) for 3 weeks (Figure 6C). These results suggested that DT treatment exerts limited adverse effects on mice, thus validating our data from cell lines. In summary, our data suggested that a low concentration (10 μM) of DT can block the phosphorylation of STAT3, the protein expression of Skp2, and the mRNA expression of STAT3 downstream genes to inhibit the migration ability of lung cancer cells. At a higher concentration (approximately 20 μM), DT can inhibit lung cancer cell proliferation.

## DISCUSSION

In ancient China, the term “lung cancer” did not exist; thus, TCM has been used to treat lung cancer–related symptoms in clinical practice, rather than to destroy lung cancer cells. In other words, TCM physicians treat patients according to a holistic consideration of the body’s condition. The most common clinical manifestations of lung cancer include cough, fatigue, and body weight loss. TCM physicians prescribe various Chinese herbal formulae or single herbs to treat such patients by differentiating syndromes according to signs and symptoms caused by lung cancer and according to TCM principles. Then danshen was widely used in different types of cancers, including prostate cancer and lung cancer, depending on the syndromes of these patients. In our previous study, we discovered that danshen can prolong the survival rate of prostate cancer patients in Taiwan through NHIRD [50]. Here, we also determined the protective efforts of danshen in advanced lung cancer by using the NHIRD. The NHIRD provided the clinical evidence that danshen could be an active agent for lung cancer. However, the limitations of this study should be noted. First, the NHI program provided reimbursements only for FHPs, which were prescribed by TCM physicians and did not include decoctions and FHPs provided by pharmacies; this might have led to the underestimation of the dosage of TCM utilization. However, this underestimation might be small because most FHPs were reimbursed. Second, we could not verify the exact dosage and duration that the study participants ingested. We presumed that all medications were taken by patients as prescribed before next visiting; this may overestimate the actual ingested dosage and duration because some degree of noncompliance is always expected. Although we discovered that the use of danshen have protective effort on advanced lung cancer patients in Taiwan through NHIRD, more serious randomized double-blind placebo-controlled trials are necessary to confirm the protective effort of danshen in advanced lung cancer patients in the further.

Abietane diterpenes that have a common ortho- or para-naphthoquinone chromophore are the major components of danshen and include IIA, TI, and DT [19]. In our data, we discovered that 3 types of abietane diterpenes, including DT, IIA and TI, all have the inhibitory effect on migratory ability of A549 cells, except salvianolic acid B (Figure 1A). These data suggested that abietane diterpenes can inhibit the migratory of A549 cells. Because the study cohort included patients depending on the code of ICD-9-CM (codes:162, malignant neoplasm of trachea bronchus and lung) from NHIRD, these patients comprised small cell lung cancer and non-small cell lung cancer. We investigated the effect of DT on both small cell lung cancer and non-small cell lung cancer cell lines and discovered that 20 μM DT can inhibit the proliferation of these lung cancer cell lines (Figure 5E and 6A). In previous study, tanshinone IIA also can inhibit the growth of small cell lung cancer H146 cells [51]. It suggested that danshen may inhibit the proliferation of both small cell lung cancer and non-small cell lung cancer cell lines. The related publication [52] showed that 5 μg/ml (about 18.889 μM) of tanshinone IIA can induce the apoptosis of A549 cells in 24 hours. The other publication [51] also reported that 5 μg/ml of tanshinone IIA can induce the apoptosis of H146 cells. In our data, we found that 20 μM DT treatments can induce apoptosis of A549 cells in 24 hours and inhibit the proliferation of H146 cells which is consistent to these previous reports [51, 52]. However, the percentages of viable cells relative to control was about 18 % when cultured with tanshinone IIA (18.889 μM) for 24 hours [52]. Our result showed that the 20 μM of DT have the similar inhibitory effect on the growth of A549 cells in 48 hours. Previous study showed that the structural difference between several tanshinones at C-15 position of furan ring resulted in the different inhibition on CYP3A (Cytochrome P450 3A) activity [53]. It is possible the different position of furan ring between DT and tanshinone IIA result in the different inhibitory effect on the proliferation of lung cancer cells.

Substantial evidence has indicated that macrophages are modulated in the tumor microenvironment to promote metastatic processes by producing many compounds, including cytokines [15]. Our previous study demonstrated that DT inhibits the tumor-promoting ability of macrophages in prostate cancer through the inhibition of the CCL2/STAT3 axis [50]. Moreover, DT may have similar effects on different cancer types. Therefore, we investigated the effects of DT on the cross talk between macrophages and lung cancer cells. In an *in vivo* study (Figure 6C, 6D), we injected human A549 cells into nude mice to obtain a xenograft animal model. Before establishing this *in vivo* model, we intended to confirm whether the effects of DT on cross talk between human lung cancer cells and murine RAW 264.7 cells (from Mus musculus) are similar to those on cross talk between human lung cancer cells and human THP-1 cells. Therefore, both human THP-1 cells and RAW 264.7 cells were used to investigate the effects of DT on macrophages [54–56]. Furthermore, we determined the direct effects of DT on the migration ability of lung cancer cells (Figure 1). In addition, we elucidated the effects of cytokines secreted from a conditioned medium of THP-1 or RAW264.7 cells treated with DMSO or DT on the migration ability of lung cancer cells (Figure 2A, 2C). Compared with Figure 2A and 2C, Figure 1 does not show cytokine secretion from THP-1 or RAW264.7 cells in the medium. Previous studies have focused on the importance of direct contact in cell–cell interactions. For example, STAT3 activation in several types of cancer cells was significantly induced by the direct coculture of macrophages and cancer cells [21, 57, 58]. To simulate the physiological interactions between macrophages and tumor cells, we used a direct mixed cell–cell coculture system to investigate lung cancer cell mobility and elucidate the signaling pathway (Figure 2B, 2D). We directly cocultured THP-1 or RAW 264.7 cells with lung cancer cells under DMSO or DT treatment for 24 hours (Figure 2B, 2D). Subsequently, the conditioned medium was collected and examined to elucidate the effects of cytokines secreted from THP-1 or RAW 264.7 cells and lung cancer cells on the migration ability of lung cancer cells. Compared with the secreted cytokines shown in Figure 2A and 2C and Figure 1, the cytokines secreted by macrophages and lung cancer cells in Figure 2B and 2D were more similar to those in the real physiological microenvironment. In summary, this study is the first to demonstrate that DT inhibits the tumor-promoting ability of macrophages in lung cancer as well as the macrophage recruitment ability of lung cancer cells.

A previous study showed that bidirectional cross talk between TAM and lung cancer cells through CCL2/CCR2 signaling is a major mechanism of the TAM-mediated promotion of lung cancer growth and metastasis [18]. Furthermore, tanshinone IIA exerts cardioprotective effects by reducing CCL2 and TGF-β1 secretion from cardiac fibroblasts [59]; however, the effects of DT on cytokines secretion from lung cancer cells and macrophages remain unclear. Therefore, we employed a human cytokine array to identify the differentially expressed cytokines in the medium of DT-treated A549 cells. Our data revealed that CCL2 and CXCL1 expression was decreased in DT-treated A549 cells, and DT inhibited CCL2 secretion from macrophages. In addition, the migration ability of A549 cells was partially rescued after the addition of 5 pg/mL of CCL2 to the conditioned medium (Figure 3J). Previous studies have reported CXCL1 as an important cytokine in lung cancer development [36, 37]. Our data suggest that CCL2 is the one of the critical cytokines controlling the migration ability of DT-treated lung cancer cells. Notably, increased IL-8 expression was observed in this study. A previous report showed a significantly higher concentration of IL-8 and CCL2 in A549 cells. Furthermore, CCL2 knockdown significantly diminished A549 cell growth without altering the IL-8 level [26]. These data suggest that CCL2 is the major cytokine controlling the signaling pathway for A549 cell growth. According to the findings of the previous report and the present study, DT may inhibit the migration of both macrophages and lung cancer cells by blocking the expression of several cytokines, and CCL2 may be one of these critical cytokines under DT treatment. However, the mechanism through which DT regulates the cross talk between these cytokines remains unclear and warrants further investigation.

Tanshinone 1 can block STAT3 Tyr705 phosphorylation in MCF-7 breast cancer cells [60]. Moreover, cryptotanshinone can inhibit STAT3 Tyr705 phosphorylation in DU145 prostate cancer cells by binding to the SH2 domain of STAT3 and blocking the formation of STAT3 dimers [61]. However, the effects of these tanshinones on the cross talk between lung cancer cells and macrophages in the microenvironment remain unclear. Our results indicate that DT can block STAT3 Tyr705 phosphorylation, which is consistent with previous studies [60, 61]. Moreover, in accordance with a previous study [40], the present study demonstrated decreased mRNA expression of several STAT3 target genes, including *CCL2, ID1, PLSCR1,* and *XBP1*. Furthermore, DT can block the expression of activated STAT3 downstream cytokines, including CCL2 and CXCL1. In addition, our ChIP assays revealed that DT treatment caused a dissociation of STAT3 from the promoter of *CCL2* gene (Figure 5D). These data suggest that DT inhibits cytokines secretion from lung cancer cells by blocking STAT3 activation. Furthermore, increased IL-8 expression was observed in this study. Some previous studies have reported that nuclear factor kappa B (NF-κB) and v-rel avian reticuloendotheliosis viral oncogene homolog A (RELA) activate IL-8 transcription [62–64]. Whole-genome mRNA expression profile results revealed that DT treatment can significantly increase the mRNA levels of *NFKB1* and *NFKB2* (p105 and p100, respectively; both belong to the NF-κB family) as well as *RELA* in A549 cells (Supplementary Figure 3A). DT treatment may increase IL-8 levels in A549 cells through the NF-κB pathway. In addition, activated STAT3 can occupy the endogenous IL-8 promoter and directly inhibit IL-8 transcription and expression [31, 32]. Our data suggest that DT may regulate IL-8 levels in A549 cells through several signaling pathways.

Studies have reported that STAT3 interacts with the Skp2 pathway to regulate the motility and invasion of cancer cells [45, 65]. Skp2 is a vital protein in lung cancer metastasis and proliferation [46–48]. More important, previous studies showed down-regulation of Skp2 can induces apoptosis in lung cancer cells [66]. Skp2 also could control p53/p300 pathway to control apoptosis [67]. In recent study, *LINC473* play the critical role for lung cancer growth [38]. In our result, we discovered 10 μM DT can inhibit the mRNA expression of *Skp2* and *LINC473* in A549 cells through mRNA array and qPCR. Moreover, we also discovered 10–20 μM DT can inhibit the protein expression of Skp2 in lung cancer cells. Although 10 μM DT only mildly suppress the growth of both A549 cells and H460 cells, DT can significantly inhibit their growth through apoptosis in higher concentration (20 μM). These results suggest that lower concentration (about 10 μM) of DT can inhibit the migration of lung cancer cells and interrupt the cross talk between lung cancer cells and macrophages through the inhibiting the STAT3 pathway and downstream proteins, including CCL2 and CXCL1. Moreover, higher concentration (20 μM) of DT can block the proliferation of lung cancer cells through apoptosis. On the other hand, our mRNA array data showed that the 30 pathways in DT-treated A549 cells were identified through KEGG enrichment analysis (Supplementary Table 2, *p* < 0.05). These pathways included transcriptional mis-regulation, apoptosis, cytokine-cytokine receptor interaction and carbon metabolism (Supplementary Table 2). These data could extend our further understanding of the underlying mechanism. Collecting together, this is a novel model demonstrating that danshen has protective efforts for the advanced lung cancers patients in Taiwan through the data of NHIRD. DT inhibit the migratory ability and the macrophage recruitment ability of lung cancer cells from the *in vitro* study. DT might be a novel anti-lung cancer agent and further prospective randomized study is warranted to validate this finding.

## MATERIALS AND METHODS

### Data source

We conducted a nationwide cohort study by using population-based data from the Taiwan National Health Insurance Research Database (NHIRD). Because National Health Insurance (NHI) is a compulsory universal program for all residents in Taiwan, the NHIRD is a comprehensive health care database that covers nearly the entire 23.7 million populations of this country. We used databases for admissions and outpatient visits, both of which included information on patient characteristics such as gender, date of birth, date of admission, date of discharge, dates of visits, and up to five discharge diagnoses or three outpatient visit diagnoses (according to International Classification of Diseases, Ninth Revision (ICD-9) CM codes). The data files also contained information on patient prescriptions, including the names of prescribed drugs, dosage, duration, and total expenditure. Following strict confidentiality guidelines in accordance with personal electronic data protection regulations, the National Health Research Institutes of Taiwan maintains an anonymous database of NHI reimbursement data that is suitable for research. As we conducted a retrospective analysis of the NHIRD and all the individual information data are de-identified, we could not obtain informed consent from included patients. This study was approved by the Institutional Review Board of Chang Gung Memorial Hospital, Chia-Yi Branch, Taiwan (201601433B1).

### Study subjects

The study cohort comprised patients diagnosed with malignant neoplasm of trachea bronchus and lung (ICD-9-CM codes:162) between 1 January 1997 and 31 December 2008. We confirmed the occurrence of lung cancer by the database of the Registry for Catastrophic Illness Patients. Patients who apply for a cancer catastrophic illness certificate are required to provide pathological reports or other supporting documents, such as laboratory and image studies. All medical records including TCM use were analyzed during the study period. For study of the advanced stages of lung cancer population, patients who had undergone lung surgery and used etoposide were excluded. A total of 60,267 patients were included in the final analyses.

### Danshen exposure and potential confounders

Finished herbal products (FHP) are the modern form of Chinese herbal remedies, of which single herb and herbal formulae are concentrated into granulated compounds, which fully reimbursed under the current NHI system of Taiwan. The list of reimbursed FHP was downloaded from the website of the NHI Administration. The corresponding drug information for each FHP include the proportions of each constituent, date and period of approval as drug, code and name of manufacturer. By using this information, we determined the original amounts of danshen, in grams, for each mixture of FHPs. Patients of the study cohort were categorized into 3 groups: those who had never used danshen or used < 30 grams of danshen after lung cancer diagnosed, those who had used ≥ 90 grams of danshen after lung cancer diagnosed, or those who had used < 90 grams and ≥ 30 grams of danshen after their lung cancer diagnosis, as per medical records. Patient demographic characteristics were investigated to determine the main independent variables affecting TCM use in the lung cancer cohort. In addition to patient gender, patient age was categorized into two groups: < = 65, > 65 years. The information of the Charlson comorbidity index (CCI) was also collected and considered as one possible confounding risk [68]. We also collected exposure information of several lung cancer treatments, including cisplatin [69, 70], carboplatin [71, 72] , erlotinib [73, 74] and gefitinib [75].

### Matched cohort

To further examine the effect of danshen use, we analyzed the data by using an alternative method. The danshen users and nonusers were frequency matched randomly by age, gender, CCI, and the year of lung cancer diagnosis at a ratio of 4:1 (nonuser versus user). Overall, 3540 insured adults (708 matched sets) were included in the matched cohort

### Statistical analysis for NHIRD

The distribution of demographic factors between the danshen users and nonusers in the study cohort and matched cohort were compared. We used the Kaplan–Meier method to estimate lung cancer cumulative incidences. The study endpoint was all-cause mortality. Cox proportional hazards models were used to compute the hazard ratios (HRs) accompanying 95% CIs after adjustment for age, gender, income, urbanization, CCI and other drug use (cisplatin, carboplatin, erlotinib and gefitinib). Two-tailed *p* = 0.05 was considered significant. Patients with a death date in the admission file and those from the beneficiaries register who were lost to follow-up were censored. All of these analyses were conducted using SAS statistical software (Version 9.4; SAS Institute, Cary, NC, USA).

### Cell culture and treatment

A549 cells (human lung adenocarcinoma cell line), H460 cells (human lung large cell carcinoma cell line), H146 cells (human small cell lung cancer cell line), H209 cells (human small cell lung cancer cell line), H1650 cells (human lung adenocarcinoma cell line), IMR-9 cells (human normal lung fibroblast), RAW 264.7 cells (mouse macrophage cell line) and THP-1 cells (human acute monocytic leukaemia cell line) were obtained from the American Type Culture Collection. The RAW 264.7 cells were cultured in Dulbecco’s Modified Eagle’s medium (DMEM) (Invitrogen Corp., Carlsbad, CA), supplemented with 10% FBS at 37°C and 5% CO2. The A549 cells, H460 cells, H146 cells, H1650 cells and THP1 cells were cultured in RPMI-1640 medium (RPMI) (Invitrogen Corp., Carlsbad, CA), supplemented with 10% fetal bovine serum (FBS) at 37°C and 5% CO2. IMR-90 cells were cultured in Minimum essential medium Eagle (Invitrogen Corp., Carlsbad, CA), supplemented with 10% fetal bovine serum (FBS) at 37°C and 5% CO2. DT was obtained from ChemFaces Natural Products Co., Ltd., China (Catalog number: CFN90162, the purity is 98% and its solubility in DMSO is > 5mg/mL). Gemcitabine hydrochloride was obtained from Sigma-Aldrich (Catalog number: G6423). Etoposide was obtained from Sigma-Aldrich (Catalog number: E1383). Erlotinib was obtained from Sigma-Aldrich (Catalog number: CDS022564). Tanshinone I (TI) was obtained from was obtained from Sigma-Aldrich (Catalog number: SI-T5330). Tanshinone IIA (IIA) was obtained from was obtained from Sigma-Aldrich (Catalog number: SI-T4952). Salvianolic acid B (SA) was obtained from was obtained from Santa Cruz (Catalog number: sc-212911). Human lung cancer cells and macrophages were cultured to 60–70% confluence prior to treatment. Medium was then replaced with fresh medium containing DT in DMSO (dimethyl sulfoxide) at the indicated concentrations. Cells treated with DMSO alone were used as untreated controls. The parental cells without treatment were used as blank control.

### Cell migration assay

Cell migration assay were performed as described previously [76]. For monoculture of human lung cancer cells, the human lung cancer cell line (A549 cells or H460 cells) (1 × 10^5^ cells/well) were plated onto upper chambers with 8-μm pore polycarbonate membrane insert in the medium without FBS. The medium with FBS was plated onto lower chambers. After treated without or with DMSO or indicated concentration of drugs for 16–24 hours, the cells migrated into the bottom are fixed, stained using 1% toluidine blue, and the numbers averaged after counting 6 randomly selected fields. For the lung cancer recruitment assay, RAW 264.7 cells or THP1 cells (1 × 10^5^ cells/well) were treated without or with DMSO or indicated concentration of drugs for 24 hours. Then, the conditioned medium or control medium were collected and plated into the lower chambers. The indicated parental human lung cancer cells (1 × 10^5^ cells/well) were plated onto upper chambers in the medium without FBS. After incubation for 16–24 hours, the cells migrated into the bottom are fixed, stained using 1% toluidine blue, and the numbers of migratory cells averaged after counting 6 randomly selected fields. For the lung cancer cells and macrophages direct mix co-culture system, the indicated lung cancer cell lines (A549 cells or H460 cells) (1 × 10^5^ cells/dish) was plated in 6 mm dish overnight. After adherent, the indicated macrophages (RAW 264.7 cells or THP1 cells) (1 × 10^5^ cells/dish) were plated into the same dish. After adherent, the direct mix coculture system were treated without or with DMSO or indicated concentration of DT for 24 hours. Then, the conditioned medium or control medium were collected and plated into the lower chambers. The indicated parental human lung cancer cells (1 × 10^5^ cells/well) were plated onto upper chambers in the medium without FBS. After incubation for 16–24 hours, the cells migrated into the bottom are fixed, stained using 1% toluidine blue, and the numbers of migratory cells averaged after counting 6 randomly selected fields. Each sample was assayed in triplicate. Each experiment was repeated at least twice.

### Wound-healing assay

Wound-healing assay were performed as described previously [77]. An IBIDI culture insert (IBIDI GmbH) consists of two reservoirs separated by a 500 µm thick wall. For the lung cancer cells wound-healing assay, an IBIDI culture insert was placed into one well of the 12 wells plate and slightly pressed on the top to ensure tight adhesion. An equal number of indicated human lung cancer cells were added into the two reservoirs of the same insert and incubated at 37°C/5% CO2. After overnight, the insert was gently removed creating a gap of ∼500 µm. After treated without or with DMSO or indicated concentrations of DT for indicated hours, the migration of human lung cancer cells was observed by using Nikon TE3000 microscope. The photographs of the same area of the wound were taken after indicated hours to measure the width of the wound. The wound closure was quantified at indicated hours post-wound by measuring the remaining unmigrated area using AlphaEase^®^FC StandAlone Software. Each experiment was repeated at least twice.

### Invasion assay

Cell migration assays were performed as described previously [78]. Cell invasion was measured using Matrigel-coated film inserts (pore size, 8-μm) fitted into 24-well invasion chambers. A549 cells (3 × 10^4^ cells) were suspended in RPMI and added to the upper compartment of an invasion chamber in the presence or absence of DT or DMSO. DMEM with FBS was added to the lower chamber. The chambers were incubated at 37°C in 5 % CO2. After 24 hours, the filter inserts were removed, and the cells on the upper surfaces of the filters were removed with cotton swabs. The invasion of human lung cancer cells was stained with crystal violet and was observed using Nikon TE3000 microscope. The numbers of invasive cells were averaged after counting 6 randomly selected fields. Each sample was assayed in triplicate, and each experiment was repeated at least twice.

### Macrophage recruitment assay

Macrophage recruitment analyses were performed as described previously [76]. The human lung cancer cells (A549 cells and H460 cells) were treated with the DT for 24 hours. The conditioned medium or control medium were collected and plated into the lower chamber of transwell plates with a 5 μm pore polycarbonate membrane insert. RAW 264.7 (1 × 10^4^ cells) cells were plated onto the upper chamber for macrophage migration assay. After incubation for 16 hours, the cells migrated into the bottom are fixed, stained using 1% toluidine blue, and the numbers of migratory cells averaged after counting 6 randomly selected fields. Each sample was assayed in triplicate. Each experiment was repeated at least twice.

### Total RNA extraction and gene chip hybridization

0.2 μg of total RNA was amplified by a Low Input Quick-Amp Labeling kit (Agilent Technologies, USA) and labeled with Cy3 (CyDye, Agilent Technologies, USA) during the *in vitro* transcription process. 0.6 μg of Cy3-labled cRNA was fragmented to an average size of about 50–100 nucleotides by incubation with fragmentation buffer at 60°C for 30 minutes. Correspondingly fragmented labeled cRNA is then pooled and hybridized to Agilent SurePrint Microarray (Agilent Technologies, USA) at 65°C for 17 h. After washing and drying by nitrogen gun blowing, microarrays are scanned with an Agilent microarray scanner (Agilent Technologies, USA) at 535 nm for Cy3. Scanned images were analyzed by Feature extraction10.5.1.1 software (Agilent Technologies, USA), an image analysis and normalization software was used to quantify signal and background intensity for each feature. Raw signal data was normalized by quantile normalization for differential expressed genes discovering. For functional assay, we used enrichment test for differential expressed genes (For most model organisms). Welgen Biotech used cluster Profiler for enrichment test for gene ontology (GO) and pathway (KEGG).

### Quantitative Real time PCR

Total RNA was extracted from lung cancer cells using the TRIzol reagent (Invitrogen, Cat. No. 15596-026) according to the manufacturer’s instructions. Reverse transcription was performed using the Superscript first strand synthesis kit (Invitrogen, Number: 11904018). Quantitative real-time PCR analyses using the comparative CT method were performed on an ABI PRISM 7700 Sequence Detector System using the SYBR Green PCR Master Mix kit (Perkin Elmer, Applied Biosystems, Wellesley, MA, USA) according to the manufacturer’s instructions. Following initial incubation at 50°C for 2 min and 10 min at 95°C, amplification was performed for 40 cycles at 95°C for 20 sec, 65°C for 20 sec and 72°C for 30 sec. Primers used were: *CCL2* forward, 5′-GTC TCT GCC GCC CTT CTG TG-3′ and *CCL2* reverse, 5′-GAC ACT TGC TGC TGG TGA TTC TTC-3′. *Skp2* forward, 5′-TTA GTC GGG AGA ACT TTC CAG GTG-3′ and *Skp2* reverse, 5′-AGT CAC GTC TGG GTG CAG ATTT-3′. *IL-6* forward, 5′-TCT GGA TTC AAT GAG GAG ACTT-3′ and *IL-6* reverse, 5′-CAG GAA CTG GAT CAG GAC TT-3′. *XBP1* forward, 5′-CCG CAG CAC TCA GAC TAC-3′ and *XBP1* reverse, 5′-TCA ATA CCG CCA GAA TCCAT-3′. *ID1* forward, 5′-CAT TCC ACG TTC TTA ACT GTT CCA-3′ and *ID1* reverse, 5′-ATT CTT GGC GAC TGG CTG AA-3′. *PLSCR1* forward, 5′-ATG ATT GGT GCC TGT TTC CT-3′ and *PLSCR1* reverse, 5′-TCC ACT ACC ACA CTC CTG ATT TT-3′. *MMP2* forward, 5′-TGG CAG TGC AAT ACC TGAAC-3′ and *MMP2* reverse, 5′-CCG TAC TTG CCA TCC TTCTC-3′. *MMP9* forward, 5′-GAC GCC GCT CAC CTT CAC TC-3′ and *MMP9* reverse, 5′-CTT GCC CAG GGA CCA CAA CTC-3′. *LINC473* forward, 5′-TTC TTT CTC TCA CTG TCT CTTT-3′ and *LINC473* reverse, 5′-TAA AAG GTC CGC CAA AGT-3′. *CXCL1* forward, 5′-TCC TGC ATC CCC CAT AGT TA-3′ and *CXCL1* reverse, 5′-CTT CAG GAA CAG CCA CCA GT-3′. *IL-8* forward, 5′-ACC ACA CTG CGC AAC ACA GAA AT-3′ and *IL-8* reverse, 5′-TCC AGA CAG AGC TCT CTT CCA TCA GA-3′. *GAPDH* forward, 5′-TGC ACC ACC AAC TGC TTAGC-3′ and *GAPDH* reverse, 5′-GGC ATG GAC TGT GGT CATGA-3′. GAPDH was used as the housekeeping gene for data normalization.

### Cytokine membrane array

The secretion medium (cell culture medium) of A549 cell lines were collected 24 hours after treating with indicated drugs. The secretion profiles of cytokines were measured using Human Cytokines array Panel A Array (R&D Systems), according to the manufacturer’s instructions. Cell culture supernatants were mixed with a cocktail of biotinylated detection antibodies. Nitrocellulose membranes (spotted with different cytokine antibodies) were then incubated the sample/antibody mixture. After several washes, streptavidin-HRP and chemiluminescent detection reagents were added, which produced light at each spot proportional to the amount of cytokine bound.

### Enzyme-linked immunosorbent assay (ELISA)

The ELISA were performed as described previously [79]. Medium was collected from monoculture of lung cancer cells or macrophages, or from co-cultures of lung cancer cells and macrophages under the treatment without or with DMSO or indicated concentrations of DT for 24 hours. Human or mouse CCL2 in medium were detected by human or mouse CCL2 ELISA kits (eBioscience, catalog number: 88-7399, 88-7391) or human IL8 ELISA kit (eBioscience, catalog number: 88-8086) according to the manufacturer’s instructions.

### Western blot analysis

For Western’s blotting, cellular extracts of the human lung cancer cell line (A549 cells or H460 cells) treated with DMSO or indicated concentrations of DT for 24 hours were prepared according to the manufacturer’s instructions. The equal amounts of protein were fractionated on a 6 or 12% SDS-PAGE and transferred to polyvinylidene difluoride (PVDF) membranes. The membranes were then blocked with 5% nonfat dried milk for 30 minutes and incubated in primary antibody for 3 hours in room temperature. The primary antibodies used were: anti-phospho(tyr705)-STAT3 antibody (*p*-STAT3)(Cell Signaling, ratio: 1:1000), anti-STAT3 antibody (Cell Signaling, ratio: 1:1000), anti-Skp2 antibody (Cell Signaling , ratio: 1:1000), anti-β-actin antibody (Santa Cruz, IB: 1:10000). The primary antibody and secondary antibody were diluted with 1% nonfat dried milk in 1× TBST (Tris-Buffered Saline Tween-20). Blots were washed by 1× TBST and incubated in horseradish peroxidase-conjugated secondary anti-mouse or anti-rabbit antibodies (Santa Cruz, ratio: 1:5000) for one hour in room temperature. After washing by 1× TBST again, protein signal was detected by chemiluminescence, using the Super Signal substrate (Pierce, Number: 34087).

### Quantification of protein level

For quantification of protein level, the images of bands from Western’s blotting were analyzed by AlphaEase^®^FC Software according to the manufacturer’s instructions. After selecting the band of each group and the background, the densities of bands were automatically calibrated by subtracting the background. The density of the group without treatment was used as the standard to calculate the ratio value of the other groups.

### Chromatin immunoprecipitation (ChIP) assay

The ChIP assay was performed in accordance with the manufacturer’s instructions (Upstate) as described previously [80]. A DNA–protein complex was sheared by sonication. A 1% portion of the sheared DNA–protein complex was kept as an input DNA sample. anti-STAT3 antibody (Cell Signaling) or normal mouse/rabbit IgG (1:500, Upstate and Sigma) were used for immunoprecipitation. Enrichment of promoter binding levels was analyzed by real-time PCR, normalized by input, and expressed as a fold increase over the blank group. Primers used for real-time PCR were as follows: CCL2 promoter, 5′- TTG GTC TCA GCA GTG AAT GGA A-3′ and 5′-AGT CAA GCA GGA GGA GGG AT-3′.

### XTT assay

The indicated lung cancer cell lines were plated at a density of 10^3^ per well, in 96-well plates, in RPMI containing 10% FBS. Once attached, the medium was replaced with RPMI containing 10% FBS. The cells were then treated with indicated drugs for 24 or 48 hours; and absorbance were measured using the XTT assay kit (Roche, Cat. No. 11465015001) according to the manufacturer’s instructions as described previously [81]. The XTT formazan complex was quantitatively measured at 492 nm using an ELISA reader (Bio-Rad Laboratories, Inc.).

### Apoptosis assay

A549 cells or H460 cells (1 × 10^6^ cells) were seeded in a 100-mm plate and cultured overnight before treatment. Then, the cells were treated with control or 10 μM of indicated drugs for 24–48 hours. Then treated cells were detected by Annexin V-FITC Apoptosis Detection Kit (Strong Biotech Corporation, Cat No.AVK250) according to the manufacturer’s instructions. In brief, at the end of the incubation period, the medium was removed. The treated cells were collected after washing by cold PBS. The supernatant was removed by centrifugation and then resuspended in FITC Annexin V binding buffer and PI by staining at room temperature in the dark for 15 min. The stained cells were analyzed by the flow cytometer BD FACSCanto (Becton Dickinson). Green fluorescence, indicative of the annexin-VFITC binding of apoptotic cells, and red fluorescence, indicative of PI uptake by damaged cells, were evaluated using logarithmic amplification and electronic compensation for spectral overlap. The amount of early apoptosis, late apoptosis, and necrosis was measured as the percentage of annexin-V positive/PI negative, annexin-V positive/PI positive, and annexin-V negative/PI positive cells, respectively.

### Immunofluorescence assay

A549 cells or H460 cells were grown on chamber slides. After treated with indicated drugs, cells were extracted with cold cytoskeleton buffer (10 mM Hepes, pH = 7.4, 300 mM sucrose, 100 mM NaCl, 3 mM magnesium chloride (MgCl_2_), 0.5% Triton X-100 (v/v) and protease inhibitor cocktail (Roche)). Cells were then fixation with 4% paraformaldehyde and permeabilized in 0.2% Triton X-100 in Phosphate buffered saline (PBS) at room temperature. Cells were immunolabelled using specific antibodies and observed on Zeiss Axioplan 2 imaging and FV300 Olympus confocal microscopy. The following antibodies were used: anti-Skp2 antibody (Cell Signaling, ratio: 1:1000).

### Mouse Xenograft Model

Mouse xenograft model were performed as described previously [82]. All procedures involving animals were approved by Animal Care and Use Committee (Approval number 2015060201) of Chang Gung Memory Hospital. Surgery was performed using sodium pentobarbital anesthesia. 10 male BALB/c-nu nude mice (18–20 g) aged 5–7 weeks were obtained from BioLASCO Taiwan Co., Ltd. and were used to build the model. A549 cells were injected (1 × 10^6^/Mouse) subcutaneously in the left and right flanks of nude mice. Two days after, mice were randomized into 2 groups, 5 mice per each group and treated intraperitoneally with vehicle (2.5% DMSO) or with 30 mg/kg DT every 2 days. Tumor volume and mouse weight were measured every 3–5 days for 3 weeks. Tumor sizes were measured and tumor volume were calculated using the formula length × width × height × 0.52. Tumor size, body weight, and mortality of the mice were monitored daily. Following 3 weeks, the mice were sacrificed.

### Statistical analyses for cell line studies

All values were the means ± standard error of mean (SEM) of the replicate samples (*n* = 3 to 6, depending on the experiment) and experiments were repeated a minimum of three times. Differences between two groups were assessed using the unpaired two-tailed Student’s *t*-test or by ANOVA if more than two groups were analyzed. The Tukey test was used as a post-hoc test in ANOVA for testing the significance of pairwise group comparisons. *P*-values < 0.05 were considered statistically significant in all comparisons. SAS9.4 was used for all calculations.

## SUPPLEMENTARY MATERIALS FIGURES AND TABLES








