# Supplementary material for: Danshen improves survival of patients with advanced lung cancer and targeting the relationship between macrophages and lung cancer cells
Source: Oncotarget. 2017 Jun 28;8(53):90925–47. doi: 10.18632/oncotarget.18767 (PMC5710895; doi:10.18632/oncotarget.18767)
Supplement: Supplementary file 1 [file oncotarget-08-90925-s001.pdf]

Danshen improves survival of patients with advanced lung cancer and targeting the relationship between macrophages and lung cancer cells

Supplementary Materials

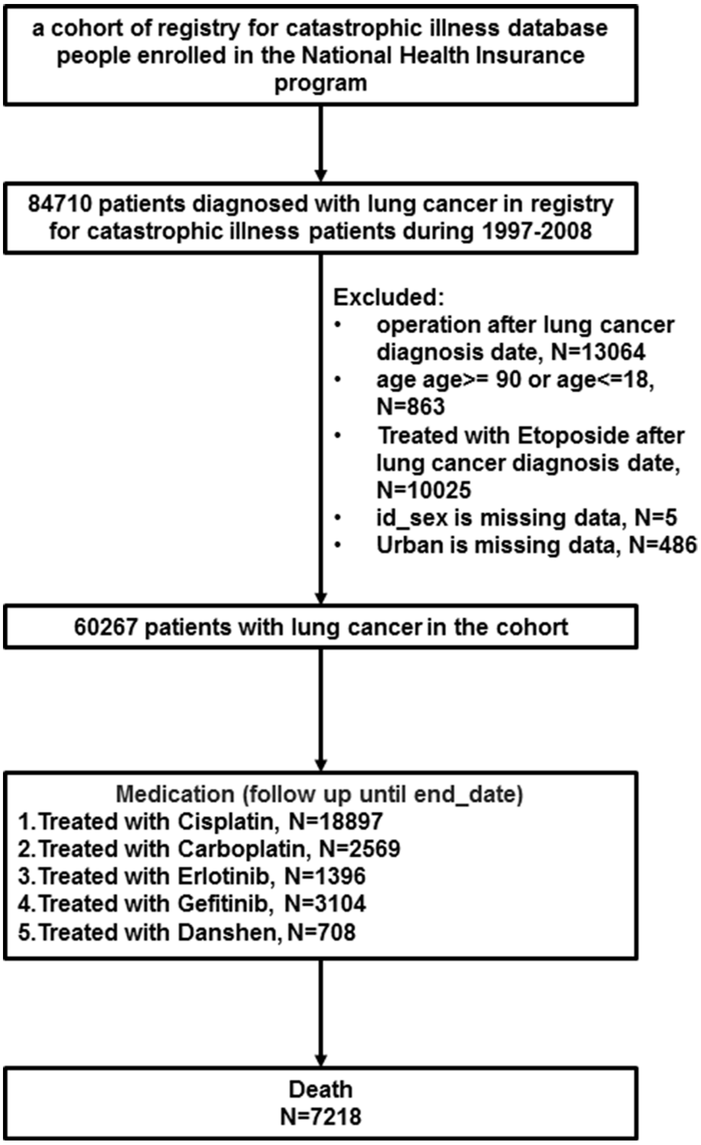

Supplementary Figure 1: Study population flowchart diagram. Of the total number of lung cancer patients registered in the NHIRD, 84710 patients were diagnosed within the years 1997–2008. After the exclusion process, the cohort contained 60267 patients.

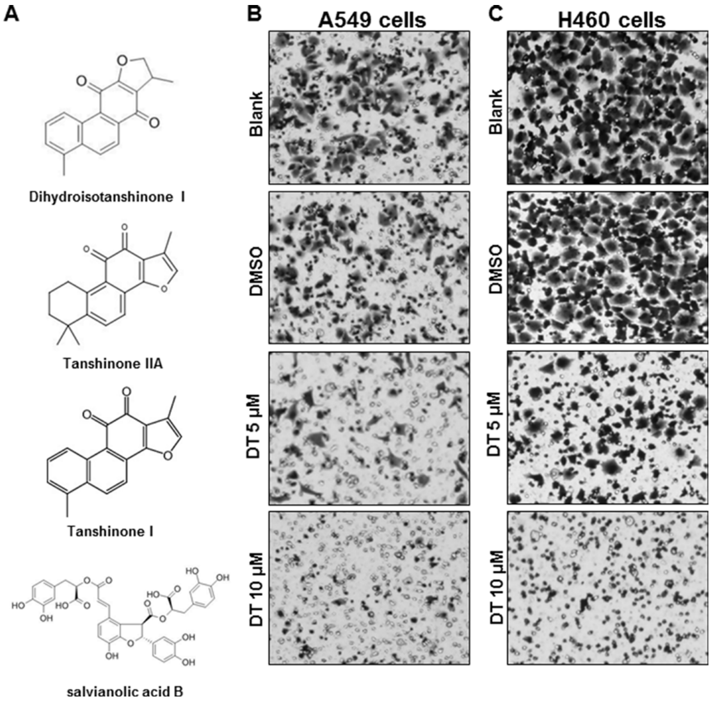
A
Dihydroisotanshinone I
Tanshinone IIA
Tanshinone I
salvianolic acid B
B
A549 cells
C
H460 cells
Blank
DMSO
DT 5 μM
DT 10 μM

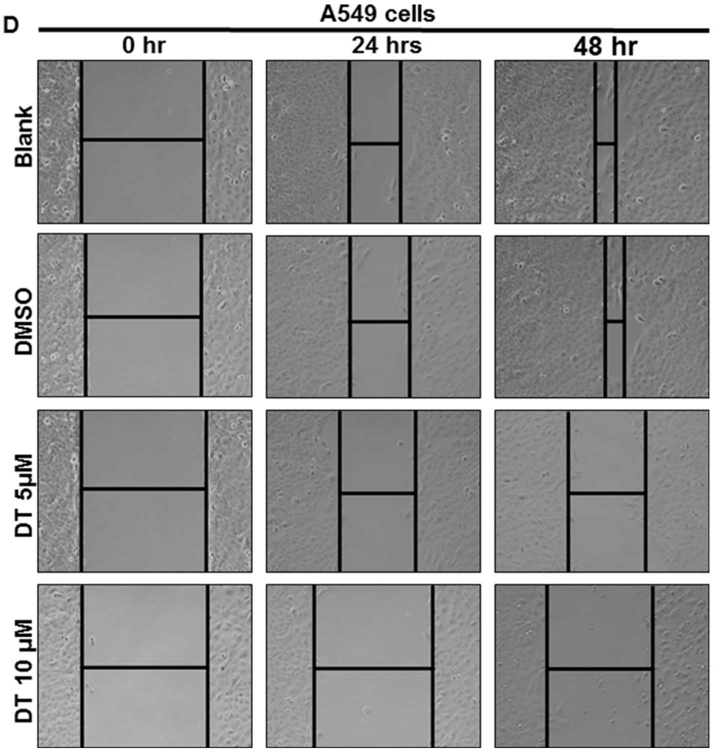
D
A549 cells
0 hr
24 hrs
48 hr
Blank
DMSO
DT 5μM
DT 10 μM

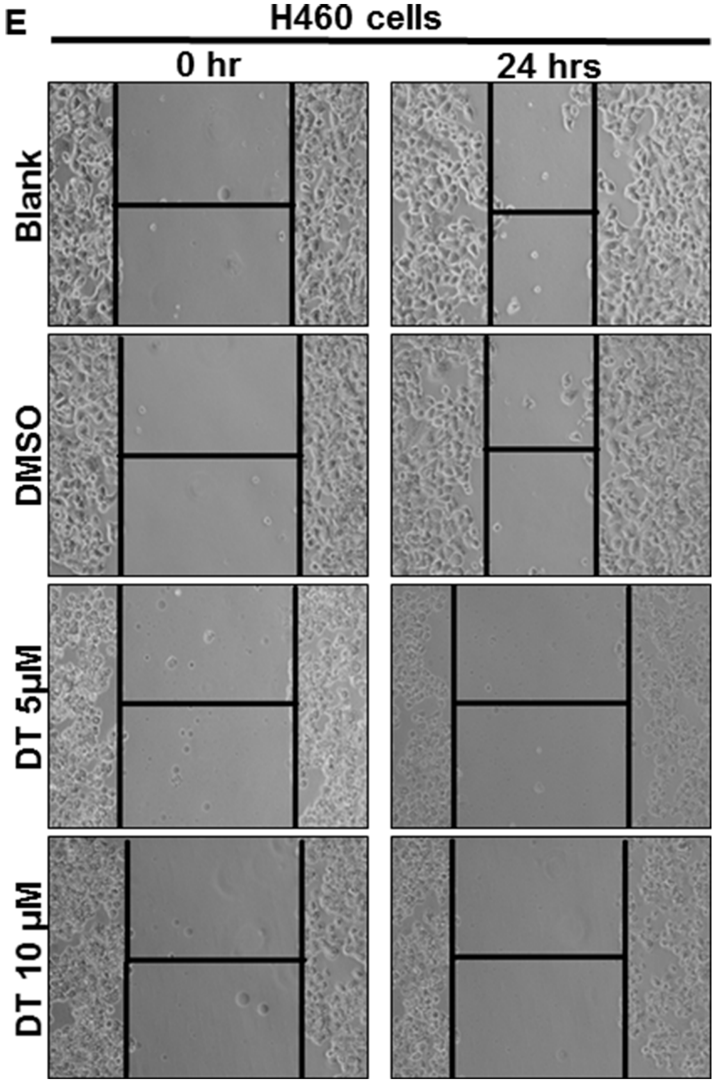

Supplementary Figure 2: DT block the human lung cancer cells migration on in vitro wound healing assay and transwell migration assay. (**A**) The structure of dihydroisotanshinone I, tanshinone IIA, tanshinone I and salvianolic acid B. (**B–E**) The migration ability of A549 cells (B) and H460 cells (C) were measured by the transwell migration assay. After treated with indicated drugs for 24 hours, the photographs (× 100) were taken. The mobility of A549 cells (D) and H460 cells (E) were measured by wound-healing assay. After treatment with indicated drugs, photographs (×100) were taken.

A

Gene Name	Description	Entrez Gene ID	Log2 Ratio
NFKB1	Homo sapiens nuclear factor of kappa light polypeptide gene enhancer in B-cells 1 (NFKB1), transcript variant 1, mRNA	4790	1.353
NFKB2	Homo sapiens nuclear factor of kappa light polypeptide gene enhancer in B-cells 2 (p49/p100) (NFKB2), transcript variant 3, mRNA	4791	0.9999
RELA	Homo sapiens v-rel avian reticuloendotheliosis viral oncogene homolog A (RELA), transcript variant 1, mRNA	5970	1.3878

B

the pathway in cancer (hsa05200)

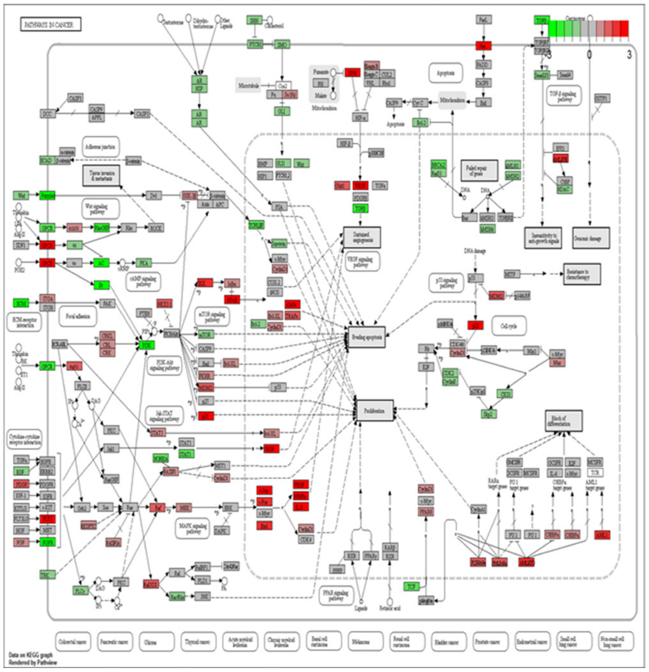

C

apoptosis pathway (hsa04210)

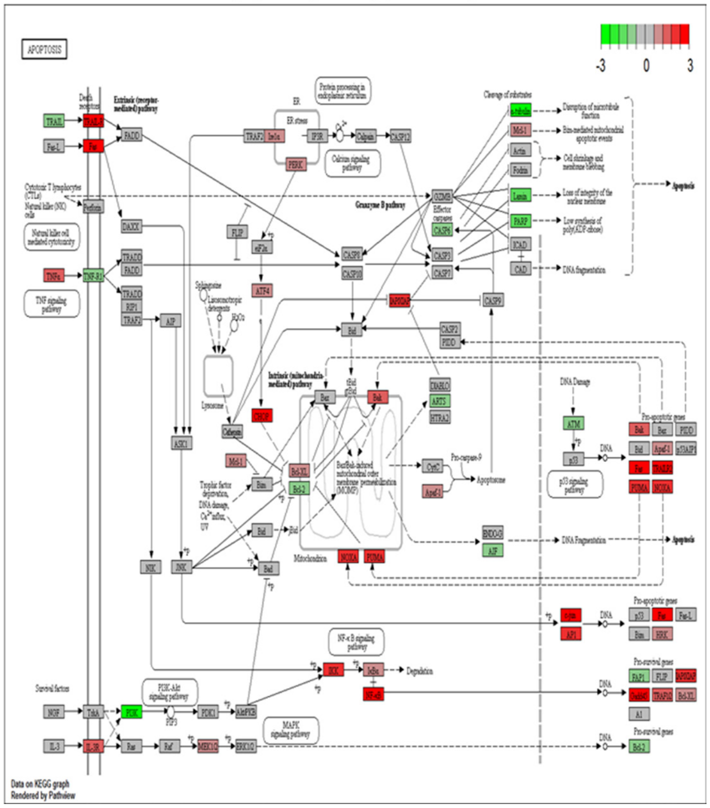

Supplementary Figure 3: The effect of DT on the whole genomic mRNA of A549 cells. (**A**) The RNA expression of NFKB1, NFKB2 and RELA were showed through mRNA array. (**B**, **C**) After KEGG enrichment analysis, the pathway in cancer (hsa05200) (B) and apoptosis pathway (hsa04210) (C) was identified. (Log2 ratio: the differential expressed level between the A549 cells treated with DMSO or DT.)

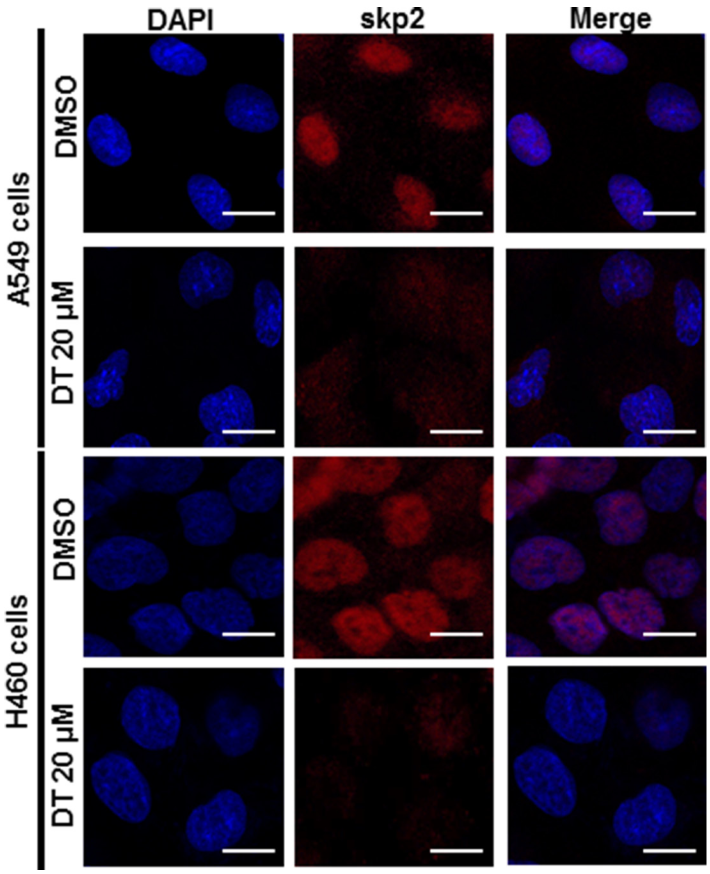

Supplementary Figure 4: DT inhibit the protein expression of Skp2. The expression of Skp2 in A549 cells or H460 cell after treatment with or without DT was detected by immunofluorescence staining with Skp2 antibodies. Cells were stained with DAPI (left panel) and anti-Skp2 antibody (right panel). All the results are representative of at least three independent experiments. Bar = 10 μm.

Supplementary Table 1: The genes which the differential expressed level between the A549 cells treated with DMSO or DT from mRNA array and lncRNA array. See Supplementary_Table 1

Supplementary Table 2: The KEGG enrichment analysis list form the mRNA array. See Supplementary_Table 2

Supplementary Table 3: The genes list which the differential lncRNA expressed level between the A549 cells treated with DMSO or DT were more than 2 folds. See Supplementary_Table 3
